# Convergence and constraint in glucosinolate evolution across the Brassicaceae

**DOI:** 10.1093/plcell/koaf254

**Published:** 2025-11-12

**Authors:** Amanda Agosto Ramos, Kevin A Bird, Annanya Jain, Gabriel Philip Sumo, Odinaka Okegbe, Lucy Holland, Daniel J Kliebenstein

**Affiliations:** Department of Plant Sciences, University of California, Davis One Shields Ave., Davis, CA 95616, USA; Plant Biology Graduate Group, University of California, Davis One Shields Ave., Davis, CA 95616, USA; Department of Plant Sciences, University of California, Davis One Shields Ave., Davis, CA 95616, USA; Department of Plant Sciences, University of California, Davis One Shields Ave., Davis, CA 95616, USA; Genetic and Genomics Graduate Group, University of California, Davis One Shields Ave., Davis, CA 95616, USA; Department of Plant Sciences, University of California, Davis One Shields Ave., Davis, CA 95616, USA; Department of Plant Sciences, University of California, Davis One Shields Ave., Davis, CA 95616, USA; Department of Plant Sciences, University of California, Davis One Shields Ave., Davis, CA 95616, USA

## Abstract

Diversity in plant specialized metabolites plays critical roles in plant–environment interactions. In longer evolutionary scales, e.g. between families or orders, this diversity arises from whole-genome and tandem duplication events. Less is known about the evolutionary patterns that shape chemical diversity at shorter scales, e.g. within a family. Utilizing the aliphatic glucosinolate pathway, we explored how the genes encoding the terminal structural modification enzyme GSL-OH evolved across the *Brassicaceae* and the genomic processes that control presence–absence variation of its products (*R*)-2-hydroxy-but-3-enyl and (*S*)-2-hydroxy-but-3-enyl glucosinolate. We implemented a phylo-functional approach to functionally validate *GSL-OH* orthologs across the *Brassicaceae* and used that information to map the genomic origin and trajectory of the locus. This uncovered a complex mechanism involving at least 3 ancestral loci with extensive gene loss across all species, creating unequal retention across the phylogenetic relationships. Convergent evolution in enantiomeric specificity was observed, where several independent species had tandem duplicates that diverged toward producing the R or *S* enantiomers. To explore potential biological differences between the enantiomers, we performed *Trichoplusia ni* larval choice assays and tested resistance against *Botrytis cinerea* in a detached leaf assay. We found that plants with the *S*-enantiomer were more susceptible to *B. cinerea* infection than to *T. ni* larval herbivory, while plants with the *R*-enantiomer seemed more susceptible to *T. ni* larval herbivory when compared to *B. cinerea*. Ultimately, we observed recurrent *GSL-OH* loss, uncovered a complex origin story for the gene, and measured the bioactivity of the enzyme's metabolic products.

## Teaser

Studying a glucosinolate enzyme across the Brassicaceae shows evolution of plant specialized metabolism involves independent gene losses, distal duplications, and reversible neofunctionalization.

## Introduction

Plant specialized metabolites mediate the myriad of plant-biotic and abiotic interactions by having a broad range of biological functions and mechanisms (Weng et al. 2021). These potential functions have recently expanded to roles as internal signaling compounds (Katz et al. 2015; Weng et al. 2016; Jia et al. 2019; Ablazov et al. 2020; Dickinson et al. 2021; Erb and Kliebenstein 2020; Singh et al. 2021; Weng et al. 2021) and storage molecules (Petersen et al. 2002; Jenrich et al. 2007; Negi et al. 2014; Qi et al. 2024), that can be recycled and reincorporated to central metabolism (Pičmanová et al. 2015; Sugiyama et al. 2021). The tasks plant specialized metabolites fulfill often involve strong and variable evolutionary pressures that contribute to shaping the wide range of specialized metabolite structural diversity (Kursar et al. 2009; Zimmermann et al. 2009; Ramírez et al. 2011; Ramos and Schiestl 2019). These roles have led to an increased interest in identifying the pathways that generate this chemical diversity or investigating the structure/function relationships in key enzymes across evolution (Weng et al. 2021). Less work has queried how well-known biosynthetic pathways evolutionarily change across closely related species.

As key pathways are identified in model species, there is a growing appreciation of the need to move beyond model species to understand how specialized metabolite pathways evolve to create diversity. Chemical analysis has shown extensive shifts in plant specialized metabolic diversity across all phylogenetic levels from order to family, genus, between and within species level (Ernst et al. 2019; Kusano et al. 2019; Huang and Dudareva 2023). This work has developed a cornerstone model suggesting that family/genus level diversity is often a result of whole-genome duplication events that enable a duplicate gene to undergo neofunctionalization and create new enzymes that form new pathways (Hofberger et al. 2013). These new enzymes can create a core structure that is then chemically modified to create extensive chemical diversity at the family to species level. At even shorter time scales, e.g. comparing closely related species or within-species variation, specialized metabolites show extensive presence/absence variation with complex patterns that make it difficult to assess if the underlying processes are parallel or convergent evolution or rampant independent gene loss (Ono and Murata 2023; Walker-Hale et al. 2025). This complicates the ability to understand the contribution of whole-genome duplications versus tandem/distal duplications in this range of specialized metabolite variation. Identifying the processes facilitating broader diversity of specialized metabolites from the species to the family level is needed to develop a deeper model of how diversity in specialized metabolism is generated.

**Figure 1.**
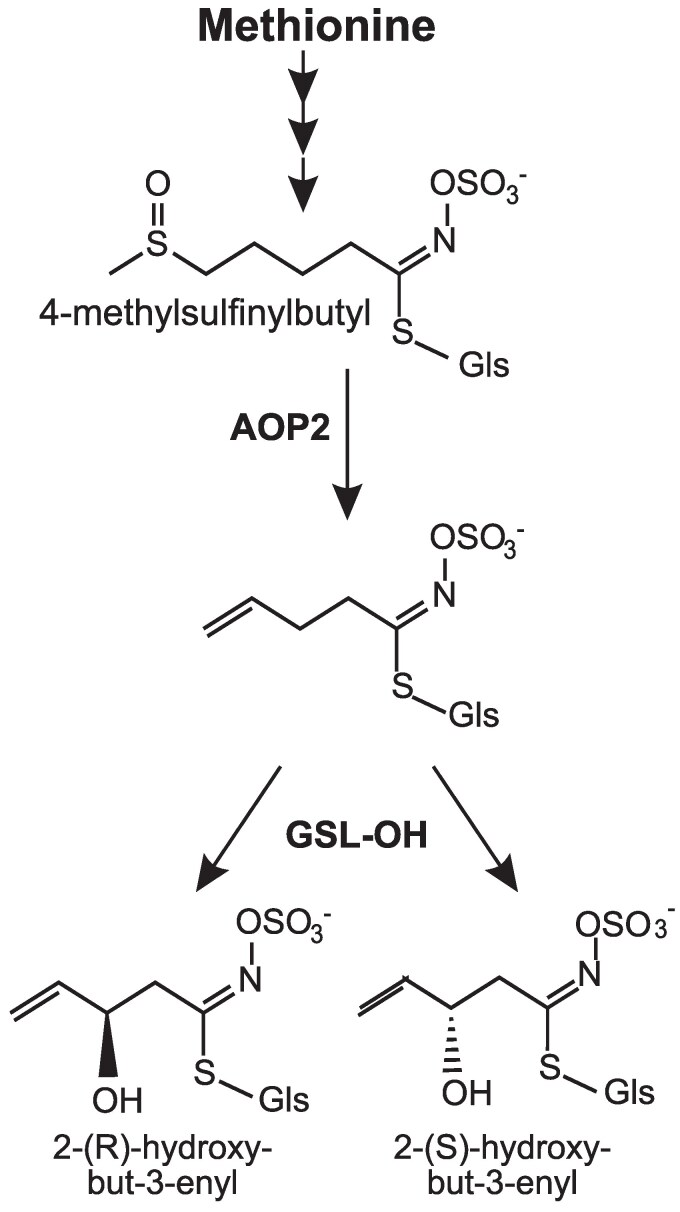
2-hydroxy-but-3-enyl glucosinolate biosynthetic pathway. In *A. thaliana,* AOP2 converts 4-methylsulfinylbutyl glucosinolate into but-3-enyl glucosinolate. GSL-OH then hydroxylates but-3-enyl, forming both enantiomeric forms of 2-hydroxy-but-3-enyl glucosinolate. Thus, its accumulation necessitates a functional AOP2 and GSL-OH.

Glucosinolates, a family of specialized metabolites primarily found in the Brassicales, are a model system to investigate the genetic and genomic processes creating chemical diversity (Hofberger et al. 2013). With 137 unique glucosinolate structures identified thus far, the pathway is considered relatively simpler and younger than other pathways that synthesize 1,000 s of different structures across numerous orders (Blažević et al. 2020). Glucosinolates are grouped by the amino acid used to create the core structure, with the methionine-derived glucosinolate pathway in the Brassicaceae family having facilitated a rapid expansion of structural diversity (Bird et al. 2025). This aliphatic glucosinolate structural diversity in *Arabidopsis thaliana* and Brassica spp. is caused by 3 main loci: *MAMs* (methylthioalkylmalate synthases), AOPs (alkenyl hydroxyalkyl producing), and *GSL-OH* (alkenyl hydroxyalkyl producing) (Kliebenstein and Cacho 2016). *MAM* encodes for *MAMs* enzymes that catalyze the initial step in the side chain elongation of methionine-derived aliphatic glucosinolates. *AOP2* and *AOP3* encode enzymes that convert short-chain aliphatic methylsulfinylalkyl glucosinolates to alkenyl- and hydroxyalkyl glucosinolates, respectively (Burow et al. 2015). The *GSL-OH* loci encode for a glucosinolate hydroxylase that can hydroxylate 4C alkenyl glucosinolates (Fig. 1). Across the Brassicaceae, these genes show epistatic presence–absence variation where production of the final 2-hydroxy-but-3-fienyl (2HB3) glucosinolate requires a MAM enzyme capable of producing a 4C side chain, an AOP2, and GSL-OH. Modular variation in these enzymes controls structural diversity by determining chain length ranging from 2 to 11 carbons, and the specific chemical modifications that occur on the side chain. Each of these loci contains extensive and complex presence/absence patterns of biosynthesis genes in *A. thaliana* and domesticated Brassica spp. that complicate parsimony interpretations. For example, initial function-to-phenotype analysis of chain elongation in *Brassica* and *Arabidopsis* identified 3 MAM enzymes (1/2/3) whose presence or absence appeared to control glucosinolate chain length through a simple evolutionary pattern across the lineage (Abrahams et al. 2020). A broader phylogenetic analysis across 40 species revealed a more complex scenario where 7 MAMs evolved through recurrent and convergent events involving tandem duplications, neofunctionalization, and independent gene losses (Abrahams et al. 2020). Additional investigations into another GLS gene family also showed a role for ectopic gene conversion to shape gene evolution (Pfalz et al. 2025). This highlights the need for comprehensive phylogenetic and functional studies to fully understand the evolution of specialized metabolism within plant families.

To develop a deeper understanding of how specialized metabolism evolves within a family, we focused on the variation controlling the production of 2HB3 glucosinolate. Metabolic analysis across the *Brassicaceae* suggests that there are potentially extensive changes in the *GSL-OH* loci leading to both presence/absence and enantiomeric variation in the reaction. *A. thaliana* also presents with intraspecies presence/absence variation; accessions that contain a functional GSL-OH accumulate a fixed ratio of 2*R* and 2*S* enantiomers of 2HB3 glucosinolate, *Brassica napus* accumulates 2*R*, and closely related *Crambe abyssinica* accumulates the 2*S* enantiomer. The side chain modification to make this compound depends on 2 enzymes, GSL-OH and AOP2. The AOP2 enzyme converts methylsulfinyl glucosinolates to alkenyl glucosinolates, e.g. 4-methylsulfinylbutyl to but-3-enyl glucosinolate, which the epistatic GSL-OH then converts to 2HB3 glucosinolate (Fig. 1). AOP3 instead converts 4-methylsulfinylbutyl into 4-hydroxybenzyl glucosinolate. Thus, if AOP3 is present and AOP2 is absent, but-3-eny glucosinolate cannot be synthesized and GSL-OH lacks the substrate needed for 2HB3 glucosinolate synthesis. As such, studying the variation in 2HB3 glucosinolate across the *Brassicaceae* and how this connects to *GSL-OH* and *AOP2* variation allows us to test the interplay of epistasis among loci, gene duplication, gene loss, and enzymatic variation in controlling glucosinolate diversity.

To empirically map how specialized metabolite diversity is evolving across shorter evolutionary time frames, we tested how the AOP2 and GSL-OH enzymatic steps evolved across the Brassicaceae. We leveraged genomic and chemical data from 46 Brassicaceae species and identified candidate *GSL-OH* and *AOPs* orthologs. Functional validation of 36 putative *GSL-OH* orthologs was conducted to map the evolution of GSL-OH function across the *Brassicaceae*. This identified novel roles for segmental and distal duplication, along with convergent shifts in stereofunctionality.

## Results

### 2HB3 glucosinolate variation in the *Brassicaceae*

**Figure 2.**
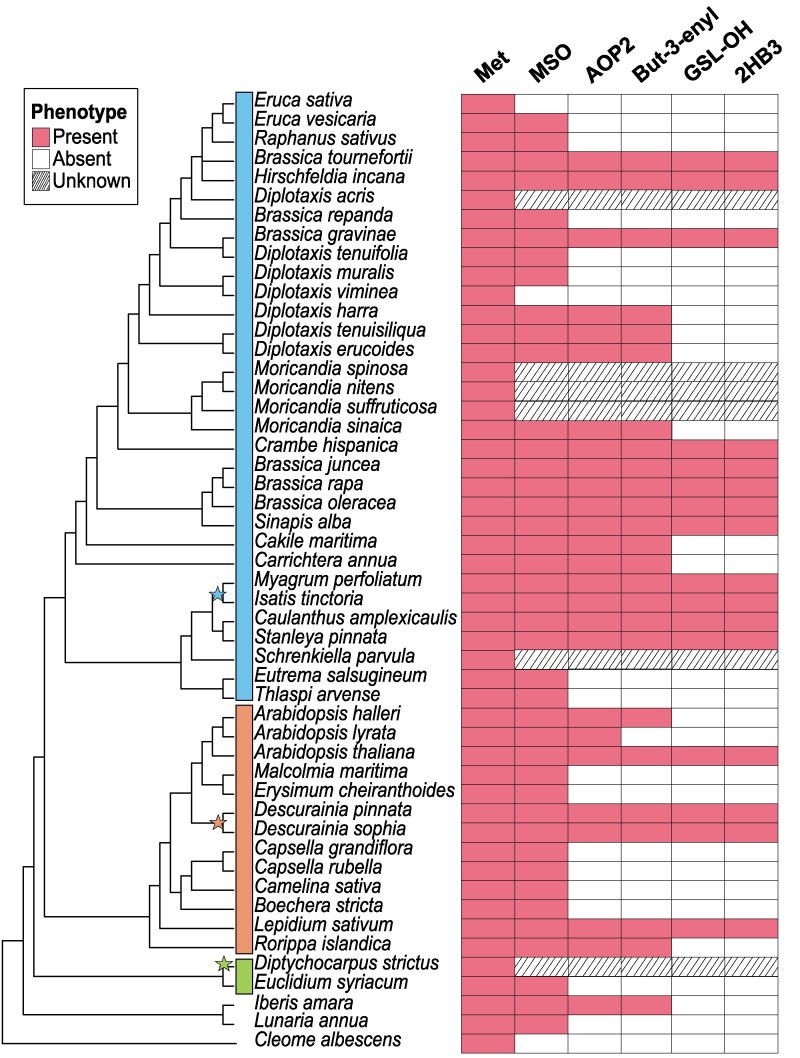
Phylogeny of 49 *Brassicaceae* species and *Cleome albescens* as outgroup, adapted from Hendriks et al. (2023). Left stripes indicate supertribes: Brassicodae (blue, lin II), Camelinodae (orange, lin I), and Hesperodae (green, lin III); unclassified taxa lack stripes. Right panel shows glucosinolate traits (multiple sources; Supplementary Table S1). Met represents methionine-derived glucosinolates, MSO stands for methylsulfinyl, AOP is for AOP2 that synthesizes but-3-enyl, and GSL-OH, which converts the latter to 2-hydroxy-but-3-enyl. AOP and GSL-OH are inferred from metabolite presence. Stars denote potential independent whole-genome duplications per Mabry et al. (2020).

To map chemical diversity, we collated the available literature on GSL-OH presence and functionality across 3 *Brassicaceae supertribes* to provide a phylogenetic context of this diversity. Supertribes Brassicodae and Camelinodae had the most representatives, while Hesperodae only had 2 representative species. The supertribes Arabodae and Heliophilodae were not included, nor was the other Brassicaceae subfamily Aethionemoideae. We identified 49 *Brassicaceae* species with published genomes that were included in a refined species phylogeny (Hendriks et al. 2023). We then collected glucosinolate content information from published datasets or in-house analysis (Fahey et al. 2001) (Supplementary Tables S1 and S2). We used the presence of 2HB3 glucosinolate to infer the existence of a functional GSL-OH ortholog. Similarly, if 2HB3 or but-3-enyl glucosinolate were reported to be present in the species, we inferred the presence of a functional AOP2. If no alkenyl glucosinolate was identified in the species, this suggests an absence of AOP2, meaning it is not possible to infer GSL-OH activity, and GSL-OH was listed as not identifiable. This allowed us to estimate GSL-OH and AOP2/3 activity across the Brassicaceae family (Fig. 2). It should be noted that the published glucosinolate profiles may lack information as they did not profile all plant tissues, at different developmental stages, and under variable environmental conditions. We were able to find glucosinolate data for 43 of the initially chosen 49 species. Of the 43 species with published glucosinolate data, 16 species contained 2HB3 glucosinolate. Of the 27 species that do not accumulate 2HB3 glucosinolate, 18 did not accumulate the precursor but-3-enyl glucosinolate, indicating an absence of the required AOP2 enzymatic activity. Nine species contained but-3-enyl glucosinolate, but did not accumulate 2HB3 glucosinolate, indicating a loss of GSL-OH activity. Mapping these events on a broader species tree suggested extensive independent presence–absence variation across the species for all major lineages (Fig. 2). However, the Brassicodae supertribe appeared to have more widespread maintenance of GSL-OH and AOP2 activities than the Camelinodae supertribe (including *A. thaliana*) (Fig. 2). For the *GSL-OH* loci, the metabolic presence–absence variation suggests at least 3 independent losses within the 40 MYA divergence time among these species. To empirically validate these gene presence/absence predictions, we proceeded to use the genome sequences to identify AOP2/3 and GSL-OH genes and to test how they map to GSL-OH activity and 2HB3 glucosinolate distribution across the *Brassicaceae*.

### AOP2/3 gene phylogeny

To systematically assess the role of *AOP2* gene variation in controlling 2HB3 glucosinolate accumulation across the *Brassicaceae*, we identified putative *AOP2* orthologs. From the initial 49 Brassicaceae, we proceeded with 46 that have publicly available genomes. This included 3 species without published glucosinolate data to test if we could recover *GSL-OH* homologs. Particularly, this included *Diptychocarpus strictus* from the Hesperodae that is under-represented in our study. Three species were not pursued, *Brassica tournefortii*, *Arabidopsis halleri*, and *Brassica gravinae*, as they are closely related to other species in the analysis. *Cleome albescens*, a member of the sister family *Cleomaceae,* was included as an outgroup (Supplementary Table S1). Using the genomic sequences, we identified *AOP2* homologues, performed sequence alignment, and constructed a phylogenetic tree using a previously developed pipeline (Steinbrenner 2024). This tree showed that *AOP2* and its tandem duplicate, *AOP3*, form a monophyletic clade with all alkenyl glucosinolate-producing species having an *AOP2* gene (Supplementary Figs. S1 and S2 and Table S2). Species with an *AOP3* tandem duplicate encode for an enzyme that synthesizes hydroxyalkyl glucosinolates, only present in *A. thaliana* and *Malcolmia maritima.* This is reflected by only these 2 species having a monophyletic *AOP3* copy. Twelve of the species do not contain any gene sequence within the *AOP2/3* clade: *Raphanus sativus*, *Lepidium sativum*, *Eutrema salsugineum*, *Erysimum cheiranthoides*, *Eruca vesicaria*, *Eruca sativa*, *Diplotaxis viminea*, *Diplotaxis tenuifolia*, *Diplotaxis muralis*, *Diplotaxis acris*, *Caulanthus amplexicaulis*, and *Brassica oleracea var. capitata*. As expected, none of these species had alkenyl, hydroxyl, nor 2HB3 glucosinolate. This suggests that presence/absence variation across the phylogenetic tree for alkenyl-derived glucosinolate production is mediated by loss of the *AOP2* gene.

### *GSL-OH* gene phylogeny

To begin a systematic assessment of GSL-OH gene distribution across the *Brassicaceae*, we used the same 46 publicly available genomes. While the *Brassicoideae* subfamily contains 5 major supertribes, the Brassicodae and Camelinodae are overrepresented in the genomes currently available (Fig. 2). As before, we used the previously developed blast-align-tree pipeline (Steinbrenner 2024) to construct a phylogenetic tree to identify all potential GSL-OH orthologs and paralogs. The resulting phylogeny contained a total of 247 genes from 46 species (Supplementary Fig. S3). Parsing the tree by divergence of the 3 *Brassicaceae* supertribes suggested at least 4 gene families within the tree.

**Figure 3.**
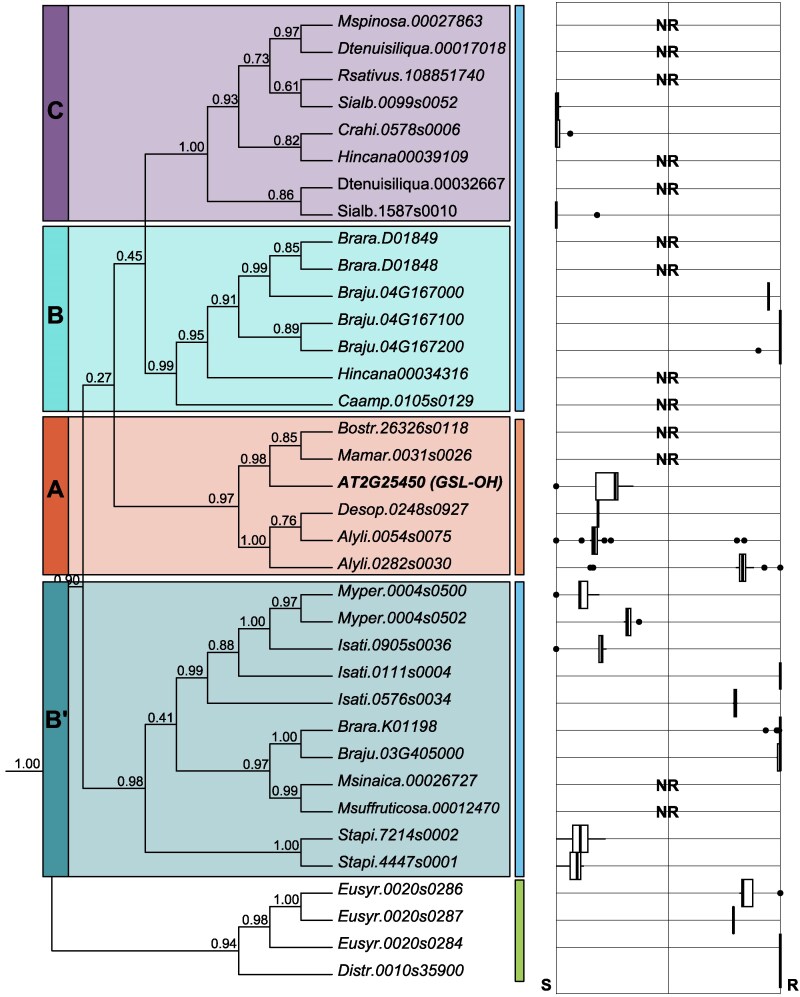
Phylogenetic and functional analysis of GSL-OH orthologs in the Brassicaceae. To the left, there is a *GSL-OH* phylogenetic tree constructed with 46 Brassicaceae genomes using the Steinbrenner lab's blast-align-tree pipeline. The *Arabidopsis* thaliana *GSL-OH* query gene is shown in bold. Shaded boxes group the genes by loci of origin. Colored lines to the right of the tree represent supertribe membership: Brassicodae (blue, lin II), Camelinodae (orange, lin I), and Hesperodae (green, lin III). Selected GSL-OH orthologs were functionally validated. To the right, the box plots show each candidate gene's measured production of either the *R-* or *S-*enantiomer of 2-hydroxy-but-3-enyl glucosinolate. Enantiomeric preference was calculated as *R*/(*R* + *S*). Data were obtained from *A. thaliana* first-generation stable transgenics expressing the respective gene. “NR” stands for no detectable reaction, indicating the enzyme did not reconstitute the GSL-OH function, thus not converting but-3-enyl into 2-hydroxy-but-3-enyl. Boxplot elements: the center line indicates the median; box limits represent the upper and lower quartiles; whiskers extend to 1.5× the interquartile range; points denote outliers beyond the whiskers.

We found 51 genes within a monophyletic clade that contained the *A. thaliana GSL-OH* (AT2G25450). Two closely related monophyletic clades were represented by the *A. thaliana* genes AT2G30830 and AT2G30840. Genetically, only AT2G25450 is required for *A. thaliana* GSL-OH activity, indicating that neither AT2G30830 nor AT2G30840 is required (Hansen et al. 2008). To validate that these were not GSL-OH encoding genes, we performed a complementation assay in an *A. thaliana* accession that accumulates but-3-enyl glucosinolate due to a natural *GSL-OH* knockout. The functional *GSL-OH/*AT2G25450 complemented the ability to synthesize 2HB3 glucosinolate in 70 independent transgenics while neither AT2G30830 nor AT2G30840 restored 2HB3 glucosinolate production (minimum 15 independent transgenic events), supporting that these closely related clades do not code functional *GSL-OH* enzymes (Supplementary Table S4). Both AT2G30830 and AT2G30840 correspond to transcripts with complete open reading frames, and the encoded proteins possess all necessary active site residues for a 2-oxoacid-dependent dioxygenase (2-ODD), with no early stop codons and are expressed in the transgenics, which suggests they likely conduct a different enzymatic reaction. Thus, we hypothesized that the monophyletic clade of *Brassicaceae* genes containing the *A. thaliana GSL-OH*, AT2G25450, is likely the functional *GSL-OH* containing clade (Fig. 3). We proceeded to functionally validate 36 of the 51 genes in this clade, representing all 3 sampled supertribes, using the above *A. thaliana* stable transgenic assay to test this hypothesis (Fig. 3 and Supplementary Table S4); reasoning for the 36 that were selected can be found in the text below and in Supplementary Table S1. These results are discussed below by phylogenetic grouping.

### Camelinodae supertribe functional validation

Given the phylogenetic proximity to the validated *A. thaliana GSL-OH* (*GSL-OH* A; Fig. 3), we began functional validation tests by focusing on all orthologs from species in the Camelinodae supertribe. Members from the super tribe Camelinodae with *GSL-OH* orthologs grouped in a single clade. We included genomes from 12 Camelinodae species; 7 species had no reported 2HB3 glucosinolate accumulation and accordingly lacked candidate genes in the *GSL-OH* clade. Their genome sequences also had no identifiable *GSL-OH* fragments, suggesting the gene has been fully lost from all 8 species. To further assess *GSL-OH* gene loss in Camelinodae, we used the high-quality genomes from the *A. thaliana* relatives, *Arabidopsis lyrata* and *halleri*. Both species have been documented to lack 2HB3 glucosinolate accumulation, and *A. halleri* also lacks *AOP2* (Stolpe et al. 2017). Neither species had any *GSL-OH* homolog, and an investigation of the syntenic region showed no residual fragments of the gene, indicating a complete loss. As a control, we selected the closest homologous gene in *A. lyrata*, AL4G25790, that resides in the AT2G30840 non-*GSL-OH* family of the tree and tested its potential *GSL-OH* activity. The gene showed no ability to rescue the accumulation of 2HB3 glucosinolate in *A. thaliana* transgenics (10 T1 events). This gene had no premature stop codons, all necessary active site residues, and was expressed in the transgenics, suggesting it is not a *GSL-OH*-like enzyme and likely performs another function. This suggests that the absence of 2HB3 glucosinolate accumulation in 7 of the 12 Camelinodae species is caused by whole gene deletions of *GSL-OH*.

To confirm the function of the 6 identified *GSL-OH* Camelinodae supertribe genes, we tested AT2G25450, Alyli.0054s0075, Alyli.0282s0030, Desop.0248s0927, Mamar.0031s0026, and Bostr.26326s0118. The genome sequenced as *Alyssum linifolium* has now been identified as *Descurainia pinnata* (Abrahams et al. 2020). *A. thaliana* accession Sha-1, which does not naturally produce 2HB3 glucosinolate as described above, was transformed with the appropriate constructs. As expected, AT2G25450 complemented the production of 2HB3 glucosinolate in all 10 independent T1 plants and T2 progeny. Alyli.0054s0075, Alyli.0282s0030, and Desop.0248s0927 led to the accumulation of 2HB3 glucosinolate in all T1 and T2 transgenic leaves (minimum of 10 independent T1 per gene). Interestingly, the 2 different *D. pinnata* genes lead to different enantiomeric ratios of 2HB3 glucosinolate. All transgenic plants containing Alyli.0054s0075 produce a ratio of 1:5(R:S) 2HB3 glucosinolate, while all transgenic plants containing Alyli.0282s0030 produce the opposite ratio of 5:1 (R:S) (Fig. 3). The 2 proteins differ in only 15 amino acids, suggesting that at least some of these changes are responsible for enantiomeric variation in *GSL-OH*.

*Boechera stricta* and *Malcolmia maritima* both have a *GSL-OH* ortholog but do not accumulate 2HB3 glucosinolate. In agreement with this, neither gene could rescue the accumulation of 2HB3 glucosinolate in any tested *A. thaliana* individual from the T1 or T2 generation (minimum of 10 independent T1s) (Fig. 3). Neither species has a functional *AOP2* nor do they accumulate the precursor, but-3-enyl glucosinolate. This suggests that the losses of *AOP2* and the epistatic *GSL-OH* occurred in a relatively close time frame. Supporting the *M. maritima GSL-OH* gene sequence containing a premature stop codon similar to the one seen in some *A. thaliana* accessions with a nonfunctional *GSL-OH* (Supplementary Fig. S5). In contrast, the *B. stricta GSL-OH* gene, Bostr.26326s0118 had no obvious genetic lesions, contained all active site amino acids required to be a functional 2-ODD (Supplementary Table S5), and the RNA accumulated in all transgenic lines, yet did not enable 2HB3 glucosinolate production. Thus, it is possible that this gene has evolved to encode an enzyme that carries out a different reaction, allowing the gene sequence to be maintained in the absence of the but-3-enyl glucosinolate precursor or GSL-OH enzymatic activity. This shows that *GSL-OH* function can be lost via both whole gene deletions and nonsense mutations.

### Brassicodae supertribe functional validation

We next proceeded to investigate the putative *GSL-OH* ortholog in the supertribe Brassicodae. We used 30 available genomes from this supertribe, most of which belonged to the Brassiceae tribe. Eleven of these species had no 2HB3 glucosinolate nor a *GSL-OH* gene, indicating complete gene losses (Figs. 2 and 3). Seventeen of the species had a gene that was positioned within the *GSL-OH* clade (Figs. 2 and 3). The distribution of the Brassicodae *GSL-OH* orthologs across the gene tree indicated that there are 3 major clades. These clades form a polytomy with the clade containing Camelinodae species, suggesting they rapidly arose near the divergence of the Camelinodae and Brassicodae supertribes (Fig. 3). We refer to these clades as Subclade *C*, Subclade *B*, and Subclade *B′*. Subclade *C* contains genes solely from species in the Brassiceae tribe. The next subclade, the *B* subclade, contains sequences from Ca*ulanthus amplexicaulis* and a few members of the Brassiceae tribe. The third subclade, *B′* subclade, contains Brassicodae species that are not in the Brassiceae tribe, along with 2 Brassiceae and appears to have originated prior to the split between the Brassicodae and Camelinodae. Most species contain a *GSL-OH* in only one subclade, while no species have genes in all 3 subclades. Three species, *Hirschfeldia incana, B. juncea,* and *B. rapa,* have genes in 2 of the subclades.

Although the Brassiceae tribe is marked by a shared whole-genome triplication (WGT) event, and we identified 3 GSL-OH-like clades within the Brassiceae species, it does not appear that the Brassiceae WGT event contributed to the origin of any of the 3 clades, nor did it expand them. Subclades *B* and *B′* both contain species that diverged prior to the WGT event, indicating their origins predate the event. Additionally, most genes appear to have rapidly lost their WGT-derived duplicates following the triplication, as all homologs from tribe *Brassiceae* species in these clades are either single-copy or tandem duplicates (Fig. 3). Subclade *C* only contains species in the tribe Brassiceae, but only one species, *Sinapis alba*, contains more than one copy, suggesting species in this clade also reduced to a single copy after the WGT event (Fig. 3 and Supplementary Fig. S4). This shows the repeated gene loss occurring within and across the GSL-OH loci. To resolve potential functional relationships within the gene tree, of the 41 Brassicodae genes in the *GSL-OH* family, we functionally validated 26 (Supplementary Tables S1 and S4).

#### *B* subclade

The *B* subclade was found in the fewest species with gene sequences in only 5 of the 46 (Supplementary Fig. S4 and Tables S1 and S4) Brassicaceae genomes. This included 2 tandem genes in *B. rapa* previously reported as potential *GSL-OH* genes; Brara.D01848 and Brara.D01849. Neither gene was functional in our validation assays, possibly due to stop codons causing both sequences to miss 50 amino acids at the *C* terminus, 2 of which are involved in 2-ODD active sites. Checking the annotated genome confirmed the early stop codons in these genes. *B. juncea* is a natural allotetraploid of *B. rapa* and *B. nigra* and contains 3 genes that group with the nonfunctional *B. rapa* genes: Braju.04G167000, Braju.04G167100, and Braju.04G167200. The encoded proteins are full-length and were all functional (Fig. 3 and Supplementary Tables S1, S4, and S5). Of the remaining tested genes in the *B* subclade, only the *Caulanthus amplexicaulis* ortholog was weakly functional, with very low amounts of but-3-enyl glucosinolate being converted to 2HB3 glucosinolate. The *Hincana00034316* gene was nonfunctional and contained active site mutations (Supplementary Table S3).

#### *C* subclade

Subclade *C* contained GSL-OH candidates from a wide range of species, including ones not known to produce 2HB3 glucosinolate. These sequences were found in only 8 of 46 total Brassicaceae species (Supplementary Fig. S4 and Tables S1 and S4). Given the presence–absence variation for the compound observed across species, we decided to test them all and explore possible explanations for the PAV observed. Of the 13 genes observed, 8 were tested, and only 3 were functional; Crahi.0578s0006, Sialb.1587s0010, and Sialb.0099s0052. Querying the other 10 genes showed that 4 genes had altered active site amino acids, likely abolishing their function (Supplementary Table S5). This subclade also had an independent change in enzyme stereospecificity, as all the functional genes in this subclade made predominantly S2HB3 glucosinolate.

#### *B′* subclade

The *B′* GSL-OH subclade had the fewest genes of Brassicae tribe species, with only *B. rapa* and *juncea*. This included the only *B. rapa GSL-OH,* Brara.K01198, gene that encodes a functional protein that results in the accumulation of the *R*-enantiomer. The close homolog Braju.03G405000 also encoded a functional enzyme that synthesized the *R*-enantiomer. In addition to these 2 Brassica sequences, the *B′* subclade is the only subclade to have sequences from *Stanleya pinnata* and species in the tribe Isatideae of Brassicaceae*: Isatis tinctoria* and *Myagrum perfoliatum*. The *S. pinnata* and *M. perfoliatum* genes were all functional with a preference to make the *S*-enantiomer (Fig. 3 and Supplementary Table S4). Interestingly, *I. tinctoria* represented the only species that contained a large expansion in the number of *GSL-OH,* with *I. tinctoria* having 9 *GSL-OH* homologous. This expansion in *I. tinctoria* and to a lesser extent *M. perfoliatum* may be linked to a neopolyploidy event (Supplementary Figs. S4 and S5) (Mabry et al. 2020). To test the function across this gene expansion, we focused on I*sati.0905s003*6, *Isati.0576s0034,* and *Isati.0111s0004*. All 3 genes could rescue 2HB3 glucosinolate production, each with a distinct enantiomeric specificity (Fig. 3).

Across all the GSL-OH subclades and species, we started with 16 species reported to accumulate 2HB3 glucosinolate (Fig. 2). Of the 16 species we tested, 14, excluding *B. tournefortii and B. gravinae,* for the reasons previously mentioned. Eleven of the 14 species accumulated 2HB3 glucosinolate and had at least one functional *GSL-OH*: *C. hispanica*, *B. juncea ssp. Integrifolia*, *B. rapa*, *S. alba*, *M. perfoliatum, I. tinctoria, C. amplexicaulis, S. pinnata, D. pinnata, D. sophioides,* and *A. thaliana.* Only *B. olereacea*, *H. incana,* and *L. sativum* did not have a functional GSL-OH while having reported 2HB3 glucosinolate accumulation. These species are known to have variable glucosinolate accumulation, including variation at the *GSL-OH* locus, indicating that the genotype sequenced for the reference genome has the absence variant (Fahey et al. 2001; Iwar et al. 2024). Only one species out of the 27 species reported not to have 2HB3 glucosinolate, *Euclidium syriacum* (Supplementary Table S1), had a functional GSL-OH. This could be a result of the genome sequence being from a different genotype than the reported glucosinolate analysis or tissue-specific expression. More broadly, this analysis shows an extensive level of gene loss both within and between species across all subclades of *GSL-OH*.

### Genomic origin of key *GSL-OH* loci

**Figure 4.**
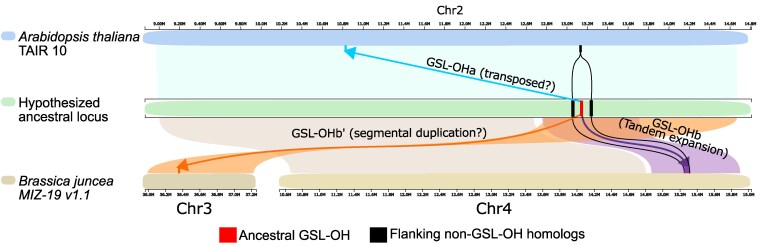
Model for the evolution of the GSL-OH locus across the Brassicaceae family. The physical positions of the *A. thaliana* GSL-OH gene, called GSL-OHa (light blue), and the two closest homologs of GSL-OH, also of the 2-ODD enzyme family (black), are represented on the top chromosome in light blue. Colored ribbons represent macrosynteny to regions on 2 chromosomes of *Brassica juncea* showing the position of 2 functional GSL-OH homologs, the tandemly duplicated GL-OHb genes (purple), GSL-OHb*′* (orange), and homologs of the 2 closely related non-GSL-OH 2-ODD genes (black). There was no detectable macrosynteny between any of these regions and GSL-OHc homologs and so they are excluded from this figure. Based on the evolutionary relationships of these genes and the patterns of macrosynteny we present a hypothetical ancestral locus in green, where an ancestral GSL-OH was nested within the 2-ODD homologs, GSL-OHb retains the ancestral position, GSL-OHb′ arose by a segmental duplication or similar mechanism, and GSL-OHa was transposed to the current location 2MB upstream of the non-GSL-OH homologs or some similar mechanism.

The phylogenetic analysis of the *GSL-OH* homologs suggested that the gene family expanded before the split between the Camelinodae and Brassicodae to create 4 distinct loci reflected as subclades in the tree (Fig. 3). To better understand how this occurred genomically, we used the syntenic relationship between the genes across the species. Comparative genomic analysis suggested that the locus likely first arose from a tandem array of 2-ODDs that currently maps to chromosome 4 in *B. juncea* and represents the *GSL-OH B* locus (Fig. 4). In the *B* locus, the functional *GSL-OH* is located between the neighboring non-*GSL-OH* 2-ODDs (AT2G30830 and AT2G30840 in *Arabidopsis thaliana* and Braju.4G163000 and Braju.4G168000 in *B. juncea*). These flanking 2-ODD genes are the most closely related genes to *GSL-OH*, suggesting that a local tandem duplication and neofunctionalization may have created the original *GSL-OH* (Fig. 4). Local macrosynteny between the *GSL-OH B* and *B′* loci indicates that the *B′* locus within the Brassicodae arose by a segmental duplication of the *B* locus. In contrast, micro- and macrosynteny analysis of the *GSL-OH C* homologs in *S. alba* and *H. incana* found no synteny to either the *GSL-OH B* or *B′* loci. This suggests that *GSL-OH C* arose by a single gene distal duplication prior to the whole-genome triplication in the Brassicaceae tribe.

Investigating synteny within the Camelinodae *GSL-O*H *A* locus shows that the *GSL-OH B* and *B′* regions from the Brassicodae map to an *A. thaliana* region on chromosome 2 from ∼12.8 to 13.7 Mb. Within these are the 2 *A. thaliana* closest *GSL-OH 2-ODD* relatives, AT2G30830 and AT2G30840, that flank *GSL-OH* in the *B* locus. However, unlike the *GSL-OH B* locus, this region does not contain the Arabidopsis *GSL-OH*. Instead, the Arabidopsis *GSL-OH* gene is located approximately 2 Mb away in a genomic block showing no macro or microsynteny to any of the *B*, *B′*, or *C* genomic regions. This suggests that, like *GSL-OH C*, the *GSL-OH A* locus in the Camelinoidae arose via an independent distal gene movement out of the neighboring *GSL-OH B* genomic position. This shows that the evolution of the gene family within these species very likely involved segmental duplications, distal duplications, and distal gene movement from an ancestral locus that arose prior to Camelinodae and Brassicodae split.

### Querying for potential differences in enantiomers biological role

**Figure 5.**
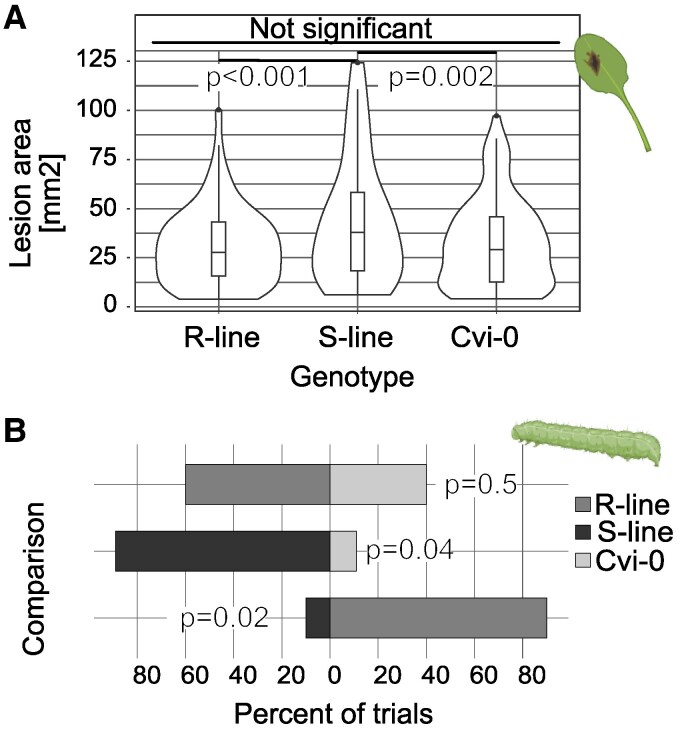
Effect of enantiomer variability: effect of enantiomer variability: biological assays were conducted with *A. thaliana* Cvi-0 as the control and background for the transgenic lines. Two lines each expressing either Alyli.0282s0030 or Alyli.0054s0075. The former produces a 5:1 (*R*:*S*) ratio of the enantiomers, the latter a 1:5 (*R*:*S*). We refer to the plants by the enantiomer primarily accumulated: R-lines and S-lines. **A)**  *B. cinerea* lesion size on *A. thaliana* Cvi-0, R-lines, and S-lines. In a detached leaf assay setup, we inoculated the leaves with spores of 1 of 6 strains of *B. cinerea*. Images of the lesion size 72-h postinfection were analyzed, and the results are shown with significance tested by ANOVA with a post hoc Tukey comparison. **B)** Dual choice test with *T. ni* on leaf disks. Three independent genotypes per gene with 3 independent plate replicates for each genotype were done for a total of 9 assays were used, except for R-lines vs Cvi-0, where 6 genotypes were used. Leaf disks were arranged in petri dishes in this combination: R-lines vs S-lines, R-lines vs Cvi-0, and S-lines vs Cvi-0. Five first instars were placed in the center of each plate, and images taken after 24 h were analyzed to determine the percentage of the leaf disk consumed. *T. ni* and leaf were designed using BioRender. Statistical tests were done using a chi-square approach.

Across all 4 subclades, GSL-OH enzymes showed diversity in the specific enantiomeric mix they produce that independently and convergently evolved across the phylogenetic tree. This raises the question of whether this variation in stereochemistry is functionally relevant for plant-biotic interactions or if it is largely neutral. Previous studies (Hansen et al. 2008) have shown the presence of the *A. thaliana* GSL-OH, which makes a 1:3 (*R*:*S*) 2HB3 glucosinolate mix and reduces *Trichoplusia ni* herbivory. To test if there is a potential difference in biological functions between the 2HB3 glucosinolate enantiomers, we used multiple independent and stable transgenic *A. thaliana* Cvi-0 lines expressing either Alyli.0054s0075 or Alyli.0282s0030 (Fig. 3). While both accumulate both enantiomeric forms, they accumulate opposite ratios of each enantiomer. Alyli.0054s0075 accumulates a 1:5 (*R*:*S*) ratio while Alyli.0282s0030 accumulates 5:1 (*R*:*S*) ratio. From here on, we will refer to these plants as S-line (Alyli.0054s0075 expressing) and R-line (Alyli.0282s0030 expressing). We selected these 2 genes as described earlier, the encoded proteins differ in 15 amino acids and the but-3-enyl glucosinolate to 2HB3 glucosinolate conversion rate is comparable; this was not the case for enzymes that resulted in the accumulation of only 1 enantiomer. We tested biotic interactions in these lines with the parental Cvi-0 accession that accumulates the precursor glucosinolate, but-3-enyl, as our wild type (WT from here on). For each transgene, 3 independent transgenic lines were chosen. These lines were tested for both *T. ni* herbivory and *Botrytis cinerea* fungal resistance. In the *B. cinerea* detached leaf assays, S-line had significantly larger fungal lesions on average across 6 different botrytis strains than did either the transgenic lines expressing R-line or the WT lines accumulating the precursor but-3-enyl glucosinolate (Fig. 5A). This suggests that S2HB3 glucosinolate is less effective at providing resistance to multiple *B. cinerea* isolates than but-3-enyl glucosinolate and R2HB3 glucosinolate. In contrast, *T. ni* choice tests showed a significant preference for consuming lines expressing R-line over S-line, with 90% of choice trials having more R than S eaten (*P*-value = 0.02) (Fig. 5B). Interestingly, the choices were altered when compared to the but-3-enyl glucosinolate-accumulating WT Cvi-0, with R-line not being statistically different from the WT (*P*-value = 0.5). When comparing S-*line* to the WT the former was more affected by herbivory (*P*-value = 0.04) (Fig. 5B). This suggests that the enantiomers and precursor have differential effects depending on the biotic attacker being tested and that the substitutions leading to amino acid sequence differences could be non-neutral. Broader, more systemic assays will be needed to assess the breadth and types of interactions that are altered by the enantiomeric differences.

## Discussion

Using the genome sequences of 46 Brassicaceae species in conjunction with empirical validation allowed us to chart the evolution of the *GSL-OH* enzyme throughout the Brassicaceae family. This showed independent and convergent presence–absence and enantiomeric variation across the major *Brassicaceae* lineages studied. Genome sequences showed that a majority of this presence–absence variation is the result of recurrent complete gene loss of *GSL-OH* with some existing pseudogenes that have either active site missense or nonsense mutations (Figs. 2 and 3, Supplementary Table S5) (Jozwiak et al. 2025). Genomic synteny analysis showed that the Brassicodae contains 3 distinct *GSL-OH* loci created by segmental and distal gene duplications that unequally partitioned across the tribe. In the Brassicodae species that had a whole-genome triplication, this would have created a potential of 9 gene copies, yet none of the Brassicoidae had more than 3 copies, while 17 had lost all 9 entirely. Species that contain a functional *GSL-OH* show evidence of independent changes in function whereby GSL-OH enzymes shift between making mainly the *R* or S enantiomers. These enantiomers had differential activity in 2 different biotic resistance assays to a fungus and an insect. This shows that simple presence/absence phenotypic variation within a metabolic trait is caused by a highly complex set of genomic processes enabling this variation.

### Duplication events in gene evolution

Evolution of specialized metabolite genes or gene families is typically associated with tandem gene or whole-genome duplication. In contrast, *GSL-OH* showed the importance of gene loss in conjunction with distal and segmental gene duplication in shaping gene family evolution (Fig. 4). The *Brassicaceae* genomes did reveal tandem gene expansion/contraction, such as the expansion within *Isatis* of *GSL-OH B′* to 9 copies. However, synteny analysis suggested that the primary driver of *GSL-OH* expansion/evolution was distal and segmental duplications that were not connected to whole-genome duplication events, also seen in *Isatis indigotica* where 3 tandem *GSL-OH* homologs can be found (Zhang et al. 2022b). We hypothesize that the original *GSL-OH* arose within the *B* position as a tandem duplicate of one of the neighboring 2-ODDs. This then underwent a segmental duplication to create the *B′*. In the Camelinodae, the *B′* gene was lost and the *B* locus distally moved to a nonsyntenic position, creating the A locus that is only present in the Camelinodae. In the Brassicodae, a further distal duplication created the *C* locus such that *B*, *B′,* and *C* were all present prior to the ancient whole-genome triplication event in the Brassiceae tribe. Distal and segmental duplications have also been found creating variation in other specialized metabolite genes, such as the origin of a novel *MAM* within the Brassicas and a novel *CYP79F* within *Boechera* (Schranz et al. 2009; Prasad et al. 2012; Abrahams et al. 2020). Broader surveys for distal and segmental duplications in phylogenetically informed species are needed to ascertain if this is a broader feature of plant specialized metabolite evolution.

### GSL-OH protein function variation

In addition to gene loss, our experimental phylo-functional approach identified an unexpected ability to shift enantiomeric specificity such that some GSL-OH proteins made predominantly the *R*, the *S*, or a mix of *R*/*S* enantiomers (Fig. 3). The enantiomeric specificity rapidly changed both between and within species. At its extreme, *I. tinctoria* had tandem gene duplications of a single locus, creating 9 copies of *GSL-OH* that could be grouped into 3 structurally similar proteins. Each group synthesized one of 3 combinations: predominantly *R*, predominantly *S*, or only *R*. This enantiomeric shift occurred across all 4 *GSL-OH* loci, such as a pair of tandem *GSL-OH* homologs in *D. pinnata* where one copy synthesized a 1:5 (*R*:*S*) ratio and the other 5:1 (*R*:*S*). A correlation of active site polymorphisms with enantiomeric specificity found no connections, suggesting that the shift may be created by altering the general shape of the active site pocket as opposed to specific moieties. This could shift the angle of oxygen attack on the but-3-enyl glucosinolate precursor. Further work is needed to ascertain how enantiomeric specificity is generated.

The enantiomeric variation raises the question of whether stereospecificity has any biological relevance. Previous data showed that the Arabidopsis 2HB3 leads to more resistance to the herbivore *T. ni,* but the work could not test if the effect depended on the enantiomer (Hansen et al. 2008). Using the *D. pinnata* genes, we could show that the *S*-enantiomer of 2HB3 glucosinolate alters resistance to a collection of *B. cinerea* isolates while the *R*-enantiomer has no effect. In contrast, *T. ni* choice tests showed a significant preference for consuming lines that primarily accumulate the *R*-enantiomer of 2HB3 glucosinolate while avoiding the lines accumulating the *S*-enantiomer of 2HB3 glucosinolate (Fig. 5B). Interestingly, the *T. ni* feeding preference altered when comparing lines accumulating the 2 enantiomers to the Cvi-0 control accumulating but-3-enyl glucosinolate. Lines accumulating the *R*-enantiomer of 2HB3 glucosinolate were fed upon at the same rate as the control. The genotypes accumulating mainly the *S*-enantiomer of 2HB3 glucosinolate were significantly preferred over but-3-enyl glucosinolate-accumulating genotypes. It is important to note that the effect of but-3-enyl versus that of 2HB3 will vary due to several factors. First, glucosinolate bioactivity results from their breakdown products. But-3-enyl and 2HB3 can ultimately be hydrolyzed into different breakdown products, differentially controlled via proteins such as the epithionitrile specifier protein and nitrile specifier proteins (Burow et al. 2009; Janowitz et al. 2009). The genes encoding these proteins show presence/absence variation across *A. thaliana* accessions. The wild-type Cvi-0 accumulating but-3-enyl, used for our biological test, has a functional ESP. Thus, but-3-enyl hydrolysis will primarily result in the formation of the epithionitrile form. The transgenic Cvi-0 accumulating 2HB3 breakdown will result in predominantly epithionitrile derivatives along with goitrin and simple nitriles. This means the results observed here could differ if a different accession lacking ESP was used. This highlights the complexity of this system and our limited understanding of the breakdown products resulting from known glucosinolates within *A. thaliana* and other Brassicaceae and their bioactivity. Russ and collaborators (2025) present a good example where they used Col-0 and Ler-1 *A. thaliana* recombinant inbred lines and measured the effect of the breakdown product formed in the presence and absence of ESP. They found 8 different products with variable effects on bacteria. Future studies in our system would greatly benefit from identifying the breakdown products formed in the but-3-enyl, R2HB3, and S2HB3 accumulating lines. Then, testing which individuals or combinations of breakdown products are responsible for the differences observed in our biological assays.

Supporting potential for different bioactivities are human studies, where a focus on the isothiocyanate catabolites showed that the goitrin (*S*-derived catabolite) affects iodine uptake by the thyroid, while epi-goitrin (*R*-derived catabolite) has no effect (Greer and Deeney 1959; Nishie and Daxenbichler 1982). In Arabidopsis Cvi-0, 2HB3 glucosinolate can be catabolized into the isothiocyanate, epithionitrile, or simple nitriles, and it remains to be determined which of these forms is the key form in mediating these bioactivities. This suggests that the enantiomers have differential effects depending on the biotic attacker being tested. Fully understanding the potential biological consequences of this variation would require systemic assays taking these genotypes and assaying them against biotic and abiotic stresses both in the field and the laboratory.

### Enantiomer accumulation: native context vs transgenic expression

The predominant 2HB3 glucosinolate enantiomer measured in the transgenic lines predominantly correlated with the reported or measured enantiomer present in the species. For example, the GSL-OH enzymes from *B. rapa* and *B. juncea* produced R2HB3, which is the 2HB3 glucosinolate form produced in *B. rapa* (Zhang et al. 2022a; Iwar et al. 2024). Similarly, *Crambe hispanica* accumulates S2HB3 glucosinolate and the *C. hispanica* GSL-OH makes solely S2HB3 glucosinolate (Finiguerra et al. 2001, Supplementary Table S4). Species like *A. thaliana* and *M. perfoliatum* that accumulate a mix of 2HB3 glucosinolate enantiomers also had matching ratios in planta and in the transgenic GSL-OH lines (Hansen et al. 2008; Quinsac et al. 1995, Supplementary Table S4). *D. pinnata*, *I. tinctoria,* and *S. pinnata* present a different scenario, as these species have GSL-OH enzymes that can produce different enantiomeric ratios. Indicating it is possible to shift the enantiomeric fraction by altering gene expression. *D. pinnata* seeds show a 5:1 (*R*:*S*) ratio that tracks the ratio in the R-line stable line. In contrast, *I. tinctoria* seed tissue accumulated a 1:1 (*R*:*S*) ratio, suggesting that synthesis may use multiple gene copies. Previous work in *Isatis indigotica* showed that 3 predicted *GSL-OH* copies are expressed at the same time with differing ratios (Zhang et al. 2022b). Future work would need to assess if *Isatis* species can shift the enantiomeric specificity across tissues or stresses. This suggests that the enantiomeric specificity in the validation assays tracks the in planta data and that some species may be able to regulate the enantiomeric ratio, while others will be fixed based on their gene complement. The intersection of presence/absence variation fixing enantiomeric ratios in some species, while gene copies enabling potential for regulated shifts in enantiomeric specificity have also been identified in solanum alkaloid production (Jozwiak et al. 2025).

### Implications

Whole-genome duplications (WGDs) have historically played a major role in enzyme neofunctionalization and the emergence of new metabolic pathways (Bowers et al. 2003; Hofberger et al. 2013), particularly over deep evolutionary timescales. However, it remains unclear how well this model applies to tandem or distal duplications over shorter timescales, such as within genera or families—where most specialized metabolic novelty arises. These shorter evolutionary windows also exhibit extensive metabolite presence/absence variation, suggesting potential parallel or convergent evolution (Ono and Murata 2023). Identifying the broader diversity of specialized metabolites created by these pathways and their unique enzymes/genes is critical to develop a deeper map of how diversity in specialized metabolism is generated across nonmodel species. This work shows that independent presence/absence variation and nonsyntenic processes can play critical roles in the evolution of specialized metabolism between closely related species. As such, when searching for homologs in nonmodel species, it is important to conduct these independently of synteny and across the entirety of all the queried genomes to identify the full complement of functional orthologs. Any syntenic or phylogenetic assumptions could lead to missed functional orthologs and alter the phylogenetic inferences. Assessing the broader potential for these observations requires more detailed phylogenetic and functional assessments of how specialized metabolism evolves within short evolutionary distances in other species and metabolites.

## Materials and methods

### Visualization of phylogeny and glucosinolate distribution

To assess our current understanding of *GSL-OH* across the Brassicaceae, we kindly received a recently published species phylogenetic tree (Hendriks et al. 2023). We narrowed it down to species that have publicly available transcriptomes and produce 2HB3 glucosinolate or if a close relative synthesizes it. For example, in lieu of *Capsella rubella*, which we studied, the tree shows *Capsella bursa-pastori*. The glucosinolate profiles were primarily obtained from (Fahey et al. 2001) and additional literature as needed. We then incorporated the glucosinolate information to ascertain if species likely contain *AOP2* activity, but-3-enyl glucosinolate, *GSL-OH* activity, and or 2HB3 glucosinolate.

### Building *AOP2/3* and *GSL-OH* gene phylogeny

To construct the *AOP2/3* and *GSL-OH* gene phylogenies, we implemented the “blast-align-tree’ pipeline developed by the Steinbrenner laboratory at the University of Washington (Steinbrenner 2024. steinbrennerlab/blast-align-tree: BAT v0.1.1 (v0.1.1) We obtained publicly available Brassicaceae coding sequences via Phytozome13 and those published in Guerreiro et al. (2023). Using these local genomes, we implemented the pipeline with the default parameter on the coding sequences. However, to account for genome duplication events, we altered the number of genes called from 5 to 10 for certain species. The tree was redrawn and visually inspected to ensure that the new genes being identified were external to the gene tree of interest. If all new genes were internal to the clade of interest, we increased the number of genes to pull from a species until no new internal genes were identified. This ensured we obtained all possible orthologs. The resulting tree was visualized using the “ggtree’ package in R studio (Yu et al. 2017).

### Cloning of candidate GSL-OH genes

From the phylogenetic analysis, we identified the most closely related *GSL-OH/*AT2G25450 candidates within each species based on a shared evolutionary pattern. The outgroup clades were defined using the AT2G30840/30 genes unable to catalyze the GSL-OH reaction. This was done regardless of the reported presence of 2HB3 glucosinolate (Fahey et al. 2001). All candidate genes were synthesized by Azenta Life Sciences or Twist Biosciences. The expression vector selected was pFAST-R05 (Shimada et al. 2010), acquired from the VIB-UGENT center of plant systems biology. The genes generated by Azenta were cloned into the pFAST-R05 plasmid with Gateway cloning and pDONR221 as the donor vector. Twist Biosciences genes were directly incorporated into the plasmid pFAST-R05. *Agrobacterium tumefaciens* was transformed with the final constructs.

### Plant transformation

The floral dip method of *A. tumefaciens*-mediated transformation of *A. thaliana* was used for T-DNA insertion (Zhang et al. 2006). The *A. thaliana* accession Shakdara-1 or Cvi-0 was used (Doody et al. 2022) as they produce but3-enyl glucosinolate, the metabolite needed for the *GSL-OH* reaction, but they do not synthesize the *GSL-OH* products, (R, S) 2HB3 glucosinolate. Most transformations were in the Shakdara-1 accessions due to a higher transformation efficiency.

### Transgenic plants segregation test

We identified transformed seeds by RFP fluorescence pFAST-R05 due to an RFP expression cassette in pFAST-R05. This allowed us to identify transformed seeds of *A.* thaliana. These were our first generation of transgenic seeds, referred to as T_1_. Seeds emitting red fluorescence when placed under a 532 nm laser attached to a camera lens were selected as positively transformed seeds and sown in the soil. After rosette formation, 40 mg of tissue was collected for glucosinolate analysis. The plants were allowed to self and the second generation of seeds was collected. These were also screened to confirm the potential enzyme activity. For each gene being tested, at least 5 independent T_1_ plants were tested by HPLC and reconfirmed in the corresponding second generation of plants (T_2_).

### Glucosinolate extraction and analysis

To estimate *GSL-OH* function in all genotypes, glucosinolates were measured as previously described (Kliebenstein et al. 2001). Briefly, the leaf material was placed in 400 *μ*L of 90% methanol. Samples were homogenized for 3 min in a paint shaker, centrifuged, and the supernatants were transferred to a 96-well filter plate with DEAE Sephadex. The filter plate with DEAE Sephadex was washed with water, 90% methanol, and water again. The Sephadex-bound glucosinolates were eluted after an overnight incubation with 110 μL of sulfatase. Individual desulfo-glucosinolates within each sample were separated and detected by HPLC-DAD, identified, and quantified by comparison to standard curves from purified desulfo-glucosinolates prepared from commercial compounds (Millipore Sigma CAS 21087-77-4 and Extrasynthese CAS: 21087-74-1). In this protocol, the R and S enantiomers of desulfo 2HB3 glucosinolates separate by about 2 min (Hansen et al. 2008).

### Transgene presence verification

To verify that the *GSL-OH* gene was being expressed in the independent T1s, leaf tissue was collected from at least 3 independent T1s per transgene and extracted RNA using Monarch Total RNA Miniprep Kit (T2010S). We then used OneTaq One-Step RT-PCR Kit (E5315S) with gene-specific primers designed to anneal at the exon–exon junction expected on each transcribed *GSL-OH* gene of interest (Supplementary Table S6). The RT-PCR product was run on a 2% agarose gel at 120 V for 30 min and visualized. If a band of the correct size was present, we concluded that the transgenic *GSL-OH* genes were transcribed into mRNA. All transgenic plants had transgene expression.

### Querying for active site mutations in nonfunctional GSL-OH genes

The protein sequences of the different *GSL-OH*-like candidate genes were queried to identify the active sites in each protein and to identify if there were missense mutations in these locations that could explain nonfunctionality. 2-ODD active site amino acids were previously defined from anthocyanidin synthase (AT4G22880) crystal structures (Wilmouth et al. 2002). This analysis, in conjunction with 2-ODD phylogenetic analysis, identified Tyr-217, Tyr142, Phe-144, Lys-213, His 232, Asp 234, His 288, Arg 298, Phe-304, and Glu-306 as the conserved active site amino acids. To identify these positions, all GSL-OH protein sequences were aligned using the ClustalOmega alignment in the msa R studio package with the default parameters (Bodenhofer et al. 2015). Files were exported and visualized in the desktop version of the software MEGA (Tamura et al. 2021). The amino acid at each conserved active site position was determined from the alignment and used to assign whether the ortholog was likely functional or nonfunctional (Supplementary Table S5 and Fig. S5).

### Identifying syntenic relationships between genes

The evolutionary trajectory of the *GSL-OH* gene family was mapped using a multistep approach necessitated by the level of presence/absence variation in this gene family across the species. To test the relationship of the Camelinodae *GSL-OH* vs the Brassicodae *GSL-OH*, by mapping macrosynteny between the *A. thaliana* TAIR 10 genome and *B. juncea ssp.* Integrifolia MIZ-19 genome v1.1 from a precompiled run of GENESPACE (Lovell et al. 2022) using the GENESPACE synteny viewer on Phytozome (Goodstein et al. 2012). This allowed focusing on the segments containing the identified *A. thaliana GSL-OH* and homologs Braju.3G405000 and Braju.4G167000. To better ascertain the relationship amongst *Brassicaceae GSL-OH* family members, the online CoGe platform was used (Lyons and Freeling 2008) to search for both micro- and macrosynteny between *S. alba* compared to the *GSL-OH* gene from the *A. thaliana* TAIR 10 and *B. juncea* MIZ-19 genomes using the SynMap (Haug-Blatzell et al. 2017) and SynFind (Tang et al. 2015).

### *Botrytis cinerea* detached leaf assay and *T. ni* choice test

Multiple independent transgenic lines in *A. thaliana* Cvi-0 of each of the Alyli.0054s0075 (S-line) and Alyli.0282s0030 (R-line) transgenes were used to test resistance to the necrotrophic fungus *B. cinerea* and resistance to the herbivore *T. ni*. The plants were grown with 16 h of light, 20 °C, and 60% humidity for 5 wk before use. At the time of selection of leaf tissue for the biological test, leaves were also sampled for metabolite analysis. Alyli.0282s0030 and Alyli.0054s0075 were selected, given their stark difference in R vs S accumulation and equal levels of 2HB3 glucosinolate production (Supplementary Table S4). For each genotype, homozygous transgenic T2 individuals were grown alongside wild-type control genotypes. For resistance assays, 6 *B. cinerea* strains—including the reference strain B05.10—were selected based on their distinctive virulence patterns on *R. sativus*, *B. oleracea*, and *A. thaliana.* These strains (2.04.12, Rose, B05.10, Apple517, KernB1, and KT) were chosen to capture variation in response to *Brassica* hosts with diverse glucosinolate compositions (Caseys et al. 2021, 2024). The use of multiple diverse genotypes of the pathogen allows assessing the consistency of the interaction across the pathogen's diversity. For each T1 transformation event, 6 homozygous T2 plants were selected, with 6 leaves chosen from each plant. Each leaf was inoculated with spores from 1 of 6 fungal strains following the protocols from Caseys et al. (2021). This resulted in 36 leaves per original transformation event, and this process was repeated for all 4 transformation events, as well as controls for each T2 plant. Pictures of the developing lesions were taken at 48-, 72-, and 96-h postinfection. The 72-h time was selected for image analysis, and infection size was digitally measured as described in Caseys et al. (2021). Statistical analysis was performed in R to obtain ANOVA values and corresponding figures following Caseys et al. (2021).

For the dual choice assays, 3 independent homozygous T2 transformants, R-line and S-line, were used. Six leaf disks per plant were used as replicates and obtained with an 8.7 mm cork borer (Fisher Scientific catalog #S15774, #4). Each experimental replicate consisted of 2 transgenic and 2 control leaf disks placed in a cross shape, such that a single leaf disk was positioned equally at the periphery of the petri dish containing 1% Phytoagar (Plant Media SKU#40100072-1) solution with a piece of Whatman filter paper on top. *T. ni* eggs were acquired from Benzon Research (Cumberland County, Pennsylvania) and hatched at 28 °C. Five first instars were placed in the center of each replicate plate, and images were taken at 24 and 48 h, with the 24-h time point selected for analysis. Reconstructed total leaf disk area and consumed leaf disk area were quantified using GIMP. Consumed leaf area was expressed as the percentage of reconstructed total leaf area per genotype within each plate. Statistical analysis was done with R.

### Accession numbers

All gene identities are listed in Supplementary Tables S2 and S3 using the gene annotation information as described in the cited genomes on October 1st, 2025.

## Supplementary Material

koaf254_Supplementary_Data

## Data Availability

The data underlying this article are available in the article and in its online supplementary material.

## Supplementary data

The following materials are available in the online version of this article.

**Supplementary Figure S1.** Gene phylogeny for *AOP2* captures *AOP3*, and *AOP1*.

**Supplementary Figure S2.** AOP2/3 clade.

**Supplementary Figure S3.** Complete GSL-OH phylogenetic tree constructed with 46 Brassicaceae genomes using the Steinbrenner lab's blast-align-tree pipeline.

**Supplementary Figure S4.** Complete *GSL-OH* clade with bootstraps.

**Supplementary Figure S5.** GSL-OH clade alignment using MUSCLE.

**Supplementary Table S1.** Phylogenetic relationship and glucosinolate chemotype of species used in this work.

**Supplementary Table S2**. Genotype and glucosinolate information for species directly assayed for seed glucosinolates.

**Supplementary Table S3.** AOP2 and AOP3 orthologues identified across all species tested.

**Supplementary Table S4**. Glucosinolate accumulation in individual plants for each GSL-OH gene being tested for function.

**Supplementary Table S5.** Active site information for each GSL-OH gene identified in this study.

**Supplementary Table S6.** Primers used to amplify each gene for DNA or RNA testing.

## Dive Curated Terms

The following phenotypic, genotypic, and functional terms are of significance to the work described in this paper:

AOP2 Gramene: AT4G03060
AOP2 Araport: AT4G03060

aop3 Gramene: AT4G03050
aop3 Araport: AT4G03050

GSL-OH Gramene: AT2G25450
GSL-OH Araport: AT2G25450
