# Supplementary material for: Convergence and constraint in glucosinolate evolution across the Brassicaceae
Source: Plant Cell. 2025 Nov 12;37(11):koaf254. doi: 10.1093/plcell/koaf254 (PMC12611242; doi:10.1093/plcell/koaf254)
Supplement: koaf254_Supplementary_Data [file koaf254_supplementary_data.pdf]

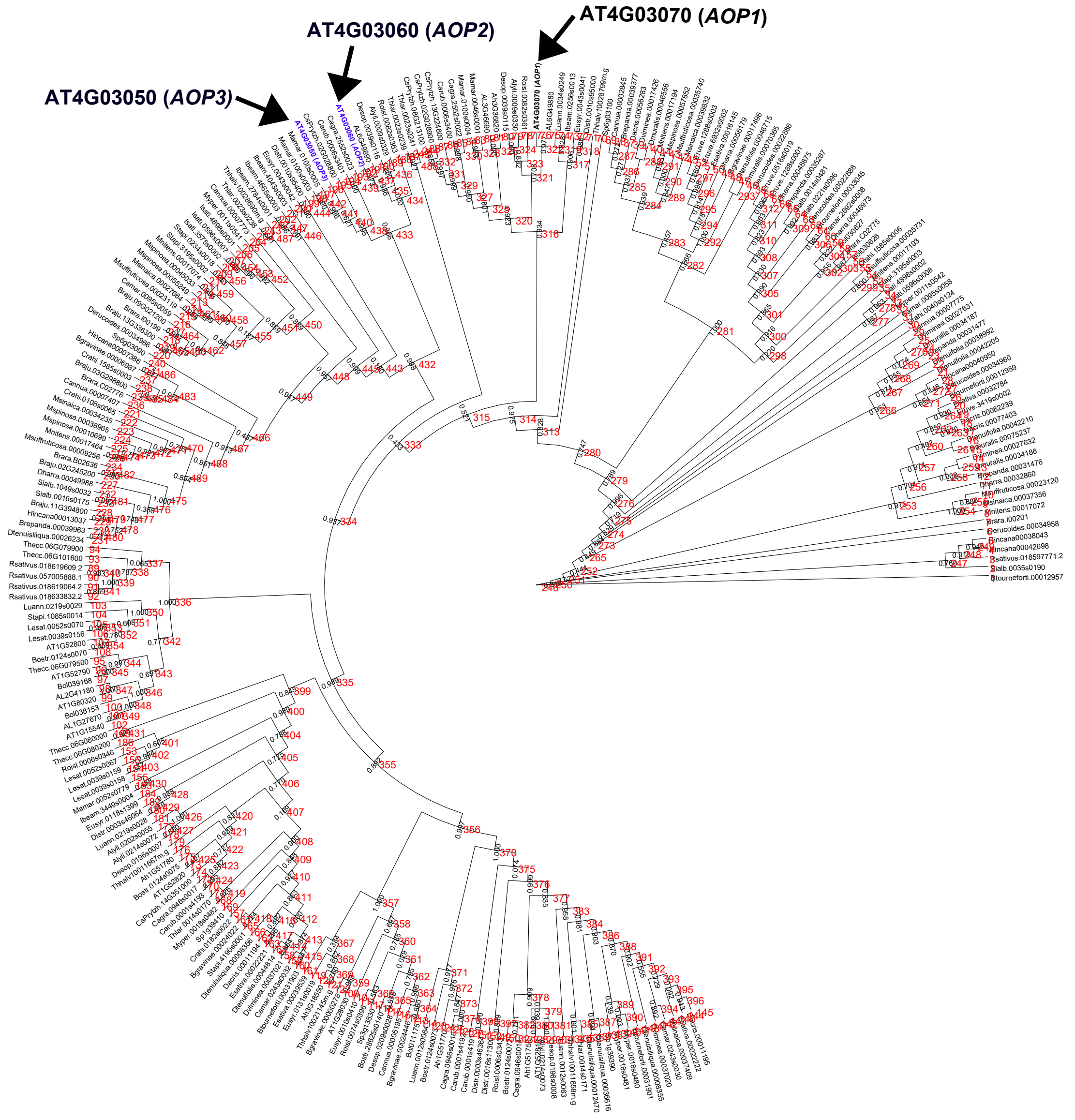

Supplementary Figure S1: Gene phylogeny for *AOP2* captures *AOP3,* and *AOP1.* Their proximity to each other makes it difficult to determine the identity of the non-*A. thaliana* homologs. This would require additional functional validation which is outside the scope of this work. However, some predictions can be made based on the glucosinolates accumulated by each species if the *A. thaliana* function enzyme function is conserved in other species.

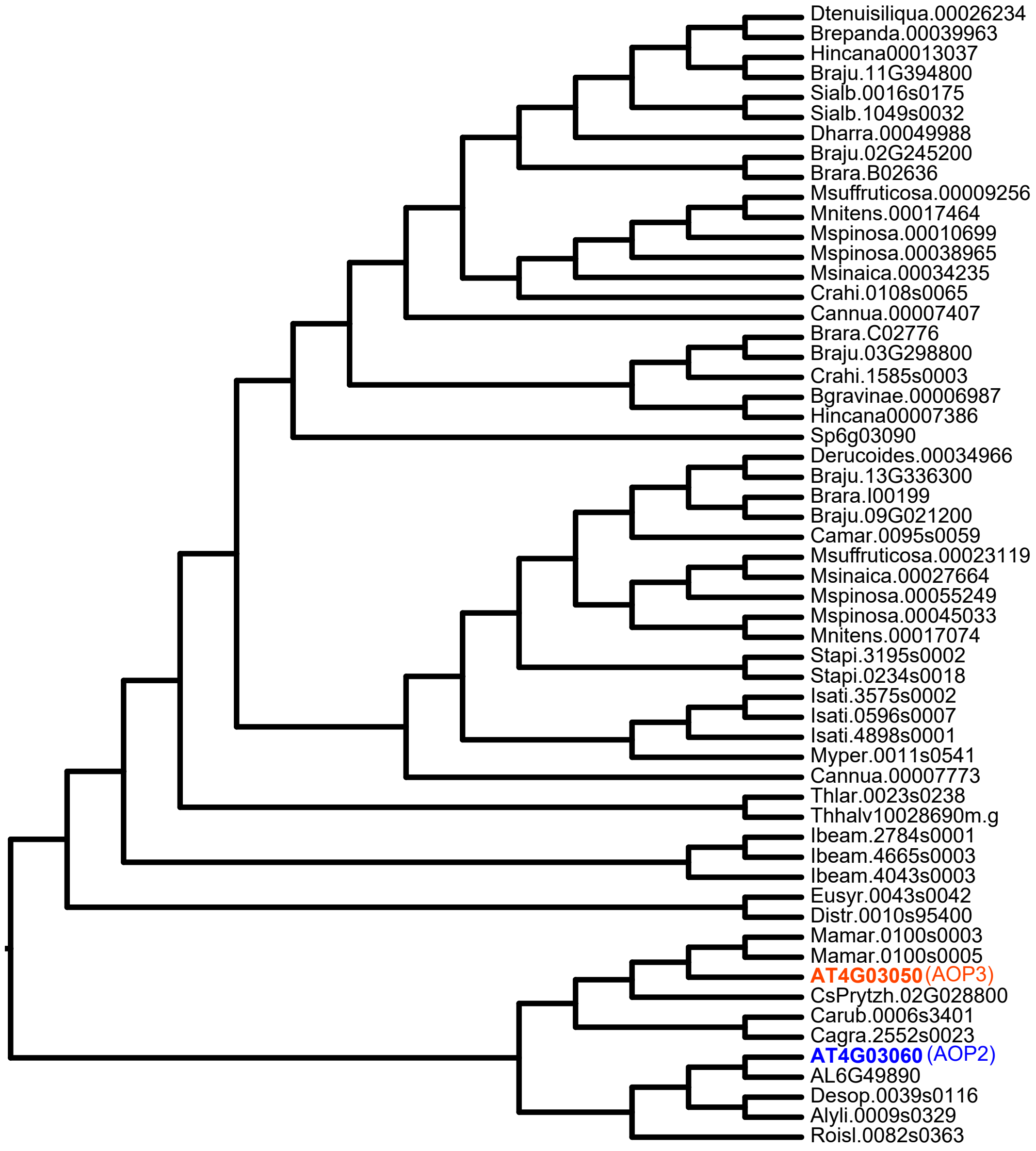

Supplementary Figure S2: *AOP2/3* clade. *AT4G3050*'s (*AOP3* colored orange) closest homologs are genes from the species *Malcolmia maritima* which like *A. thaliana* accumulates the enzyme's product, hydroxyalkyl glucosinolates. It also lacks an *AOP2* (colored blue) homolog resulting in no accumulation of alkenyl glucosinolate. The remaining homologs and how they relate to the species studied for *GSL-OH* can be found in Supplementary table 2.

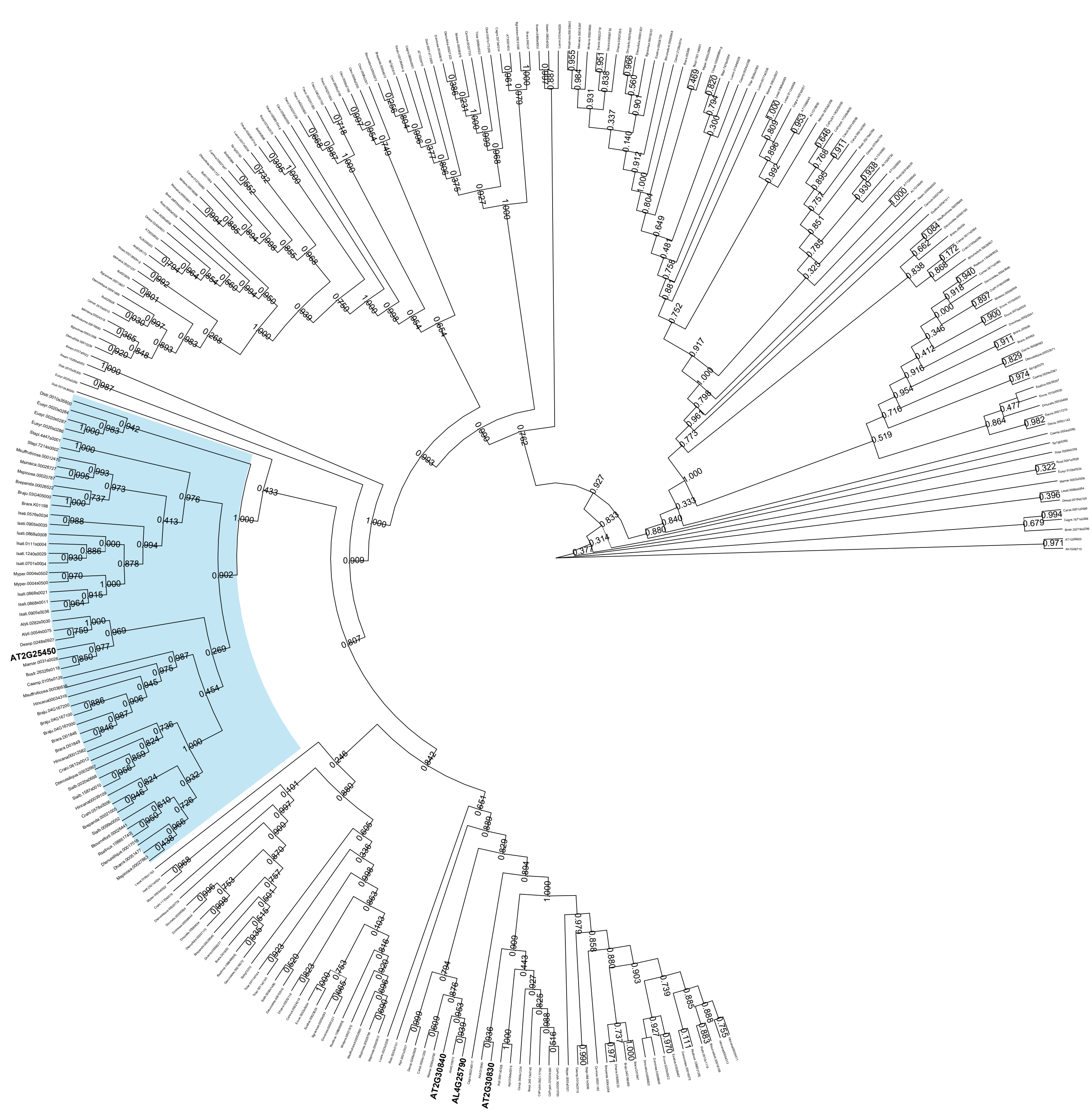

Supplementary Figure S3: Complete GSL-OH phylogenetic tree constructed with 46 Brassicaceae genomes using the Steinbrenner lab's blast-align-tree pipeline. Area highlighted in blue is the GSL-OH clade. Bold genes, *AT2G30840*, *AT2G30830*, *AL4G25790*, are the closest *Arabidopsis* genes and are non-functional. *Brassica tournefortii* was excluded from downstream analysis due to poor annotations.

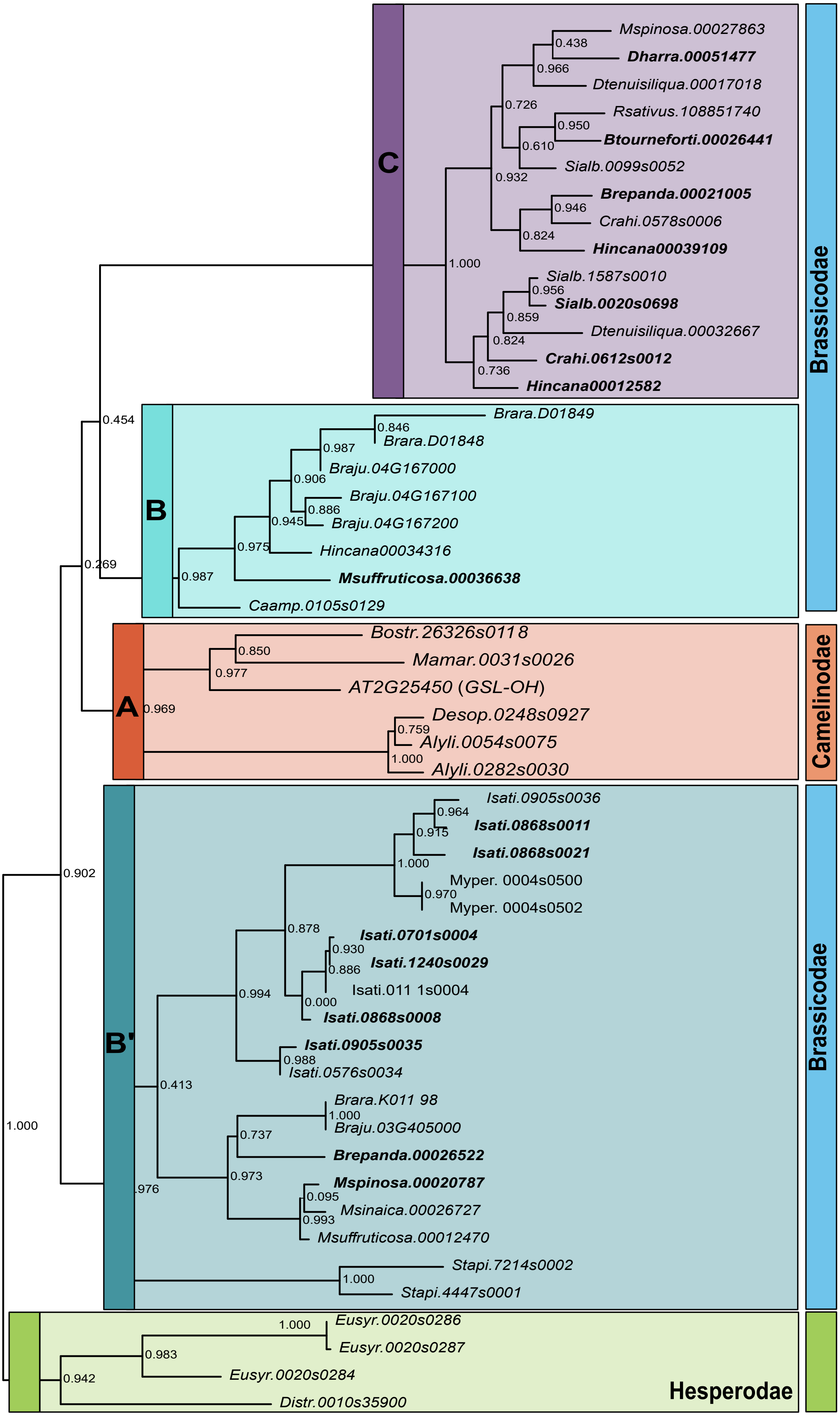

Supplementary Figure S4: Complete *GSL-OH* clade with bootstraps. Based on function and synteny analysis (Fig. 4) we identify the ancestral loci *A, B, B′,* and *C.* Due to the limited number of species in *Hesperodae* we did not assign it a group at this time. *Isatis tinctoria* has experienced a gene expansion many of them with nearly identical protein sequences. Due to financial and spatial constraints not all genes were tested. Explanations on how the choices were made can be found in the text and supplementary tables. Genes in bold were not functionally validated.

```
Alyli.0054s0075|Alyli.0054s0075.1   1   ------------------MESLDT-MPGKYDRASEVKAFDETKTGVKGLVEAGITKIPRIFHNPLVTVPN
Alyli.0282s0030|Alyli.0282s0030.2   1   ------------------MESLDT-RTEKYDRASEVKAFDETKTGVKGLVEAGITKIPRIFHNPLVTVPN
AT2G25450|AT2G25450.1               1   -------------------------MAENYDRASELKAFDEMKIGVKGLVDAGVTKVPRIFHNPHVNVAN
Bostr.26326s0118|Bostr.26326s0118.1 1   -------------------------MTGDYDRAGEIKAFDEMKIGVKGLVDAGITKIPRIFHNPDAAITN
Braju.04G167000.1                   1   -------------------------MAGTFDRADEVKAFDEMKIGVKGLVDAGITKIPRIFHNPQATVTN
Braju.04G167100.1                   1   -------------------------MVGTLNRAGDVRAFDEMKIGVKGLVDSGIKKIPLIFHNPQATVTN
Braju.04G167200.1                   1   -------------------------MSGTFDRADEVKAFDEMKIGVKGLVDAGIKKIPLIFHNPQATVTN
Brara.D01848|Brara.D01848.1         1   -------------------------MAGTFDRADEVKAFDEMKIGVKGLVDAGITKIPRIFHNPQATVTN
Brara.D01849|Brara.D01849.1         1   -------------------------MSGTFDRADEVKAFDEMKIGVKGLVDAGIKKIPLIFHNPQATVTN
Brara.K01198|Brara.K01198.1         1   ----------------------------MLDRASEVKTFDEMKMGVKGLVEAGMTKIPRIFHNPLASVTT
Brepanda.00021005                   1   ------------------MENK---MVGAYDRAGDVKAFDEMKTGVKGLVDAGIKEIPRIFHNPLVAVKN
Brepanda.00026522                   1   ------------------MESKGT-AVSILDRATEVKKFDEMKMGVKGLVDAGITKIPRIFHNPHAAVTN
Caamp.0105s0129.1                   1   -------------------------MAGTFDRACEVKAFDELKIGVKGLVDAGITKIPRMFHNPHITVTN
Crahi.0578s0006|Crahi.0578s0006.1   1   ------------------MENK---MVGAYDRAGDVKAFDEMKTGVKGLVDAGIKEIPRIFHNPLVSVKN
Crahi.0612s0012|Crahi.0612s0012.1   1   ------------------MENT---MVGAYDRAAEVKEFDEMKTGVKGLVDAGIKEIPRIFHNPLVSVEN
Desop.0248s0927|Desop.0248s0927.1   1   ------------------MGSLGT-MPEKYDRASEVKAFDETKTGVKGLVEAGITKIPRIFHNPLVTVPN
Dharra.00051477                     0   ----------------------------------------------------------------------
Distr.0010s35900.1                  1   ------------------MESKDKEAAATFDRASEVKAFDEMKTGVKGLVDAGITQIPRIFHNPLAKLTN
Dtenuisiliqua.0003266               1   ------------------MENT---MVEAYDRAAEVKEFDEMKTGVKGLVDAGITQIPRIFHNPLVSVKN
Dtenuisiliqua.00017018              1   ------------------MENK---MVGAYDRAAEVKAFDEMKTGTKGLVDAGIKEIPRIFHNPLVSVEN
Eusyr.0020s0284.1                   1   ------------------MESKDT-VAATFDRAGEVKAFDEMKTGVKGLVEAGITQIPRIFHHQLHTLTN
Eusyr.0020s0286.1                   1   ------------------MESKDR-VAATYDRAGEAKAFDEKKTGVMGFVEAGITQIPRIFHHPVHTLTN
Eusyr.0020s0287.1                   1   ------------------MESKDR-VAATYDRAGEAKAFDEKKTGVMGFVEAGITQIPRIFHHPVHTLTN
Hincana.00012582                    1   ----FNKKNKQKKKNRRKMENT---MVGAYDRAAEVKEFDEMKTGVKGLVDAGITEIPRIFHNQLVAVTN
Hincana.00034316                    1   -------------------------MVGTFDRVDEVKAFDEMKIGVKGLVDAGITKIPRIFHNPHATVTN
Hincana.00039109                    1   SQSFDSTTRRKIPTEEEKKEDMENQMIGAYDRAADVKAFDETKTGVKGLVDAGITEVPRIFHNPLVAVEN
Isati.0111s0004|Isati.0111s0004.1   1   ------------------MESKGT-MVGTLDRADDVKTFDEMRMGVKGLVDAGITKIPRIFHNPQAKVTN
Isati.0576s0034|Isati.0576s0034.1   1   ------------------MESKGT-MVGTLDRADEVKTFDEMRMGVKGLVDAGITKIPRIFHNPQATVTN
Isati.0905s0036|Isati.0905s0036.1   1   ------------------MESKGT-MVGALDRAVELKAFDETRLGVKGLVDAGITKIPRIFHNPQAKVTN
Mamar.0031s0026|Mamar.0031s0026.1   1   -------------------------MEENYDRASDVKAFDKMKIGVKGLFEAGITKIPCIFHNPHVTVTN
Msinaica.00026727                   1   ------------------METKST-VVGMLDRVSEVKTFDEMKMGVKGLVDAGITKIPRIFHNPHAAIRY
Mspinosa.00020787                   1   ------------------METKST-VVGMLDRVSEVKTFDEMKMGVKGLVDAGITKIPRIFHNPHAAVRN
Mspinosa.00027863                   1   -------------------------MAGAYDRAAEVKAFDEMKTGAKGLVDAGITKIPRIFHNPLVTVEN
Msuffruticosa.00012470              1   ------------------METKST-VVGMLDRVSEVKTFDEMKMGVKGLVDAGITKIPRIFHNPHAAVRN
Msuffruticosa.00036638              1   -------------------------MAGTLDRADEVKAFDEMKIGVKGLVDAGIKKIPRIFHNPHATVTN
Myper.0004s0500|Myper.0004s0500.1   1   ------------------MESKGT-MVGTLDRADELKAFDEMRLGVKGLVDAGITKIPRIFHNPQAKVTN
Myper.0004s0502|Myper.0004s0502.1   1   --MHTEKLQKEREKQSKTMESKGT-MVGTLDRADELKAFDEMRLGVKGLVDAGITKIPRIFHNPQAKVTN
Rsativus.108851740                  1   ------------------MENK---MVGAYDRAAELKAFDEMKTGTKGLVDAGITEIPRIFHNPLVAVEN
Sialb.0020s0698.1                   1   ------------------MEKT---MVGAYDRAAEVKEFDEMKTGVKGLVDAGITEVPRIFHNPLVSVKN
Sialb.0099s0052.1                   1   ------------------MESK---MVEAYDRAAEVKAFDEMKTGAKGLVDAGITEIPRIFHNPLVAVEN
Sialb.1587s0010.1                   1   ------------------MEKT---MVGAYDRAAEVKEFDEMKTGVKGLVDAGITEVPRIFHNPLVSVKN
Stapi.4447s0001|Stapi.4447s0001.1   1   ------------------MVGTSCTMVGTLDRADELKTFDEMKIGVKGLVDAGITKIPRIFHNPHATVTN
Stapi.7214s0002|Stapi.7214s0002.1   1   ------------------MVGTSCTMVGTLDRADELKTFDEMKIGVKGLVDAGITKIPRIFHNPHATDPN

Alyli.0054s0075|Alyli.0054s0075.1   52  P-RPTSLMSLPTIDLGGGVFESKAMRENVVNKVKDAAEKFGFFHAINHGIPLELMEKMKDGIRGFHEQDP
Alyli.0282s0030|Alyli.0282s0030.2   52  P-RPTSLMTLPTIDLGGGVFESKAMRENVVNKVKDAAEKFGFFHAINHGIPLDLLEKMKDGIRGFHEQDP
AT2G25450|AT2G25450.1               46  PKPTSTVVMIPTIDL-GGVFESTVVRESVVAKVKDAMEKFGFFQAINHGVPLDVMEKMINGIRRFHDQDP
Bostr.26326s0118|Bostr.26326s0118.1 46  PKLSSTVVMIPTIDLGGGVFESAVVRESVVAKVKDAMEKFGFFKAINHGVPLDVMEKMRNGIRGFHEQDT
Braju.04G167000.1                   46  P-KPPSTLTIPTIDLRGGVFESTVTRKEVTEKVKDAMEKFGFFQAINHGIPLEVMEKIKEGIRAFHAQDP
Braju.04G167100.1                   46  P-KPPSTLTIPTIDMGGGVFESTVTRKEVTEKVKDAMEKFGFFQAINHGIPLEVMEKMKDGIRAFHAQDP
Braju.04G167200.1                   46  P-KPPSTLTIPTIDMGGGVFESTVKRKEVTEKVKDAMEKFGFFQAINHGIPLEVMEKMKDGIRAFHAQDP
Brara.D01848|Brara.D01848.1         46  P-KPPSTLTIPTIDLRGGVFESTVTRKEVTEKVKDAMEKFGFFQAINHGIPLEVMEKIKEGIRAFHAQDP
Brara.D01849|Brara.D01849.1         46  P-KPPSTLTIPTIDMGGGVFESTVKRKEVTEKVKDAMEKFGFFQAINHGIPLEVMEKMKDGIRAFHAQDP
Brara.K01198|Brara.K01198.1         43  P-KPPSTVRIPTIDLRGGVFDSEVTRQSVVAKVKEAMEKFGFFQAINHGIPLHVMEEMEAGIRGFHGQDP
Brepanda.00021005                   50  Y-KPFSEVRIPSIDFGGGVFESPVTRESVVAKVKDAVEKYGFFQSVNHGFPLDLMQRMRDGVRAFHDQDP
Brepanda.00026522                   52  P-KPSSTVRIPTIDLGGGVFDTKVTRESVVAKVKDAMGKFGFFQAINHGIPLHVVEKMKAGVRGFHEQDS
Caamp.0105s0129.1                   46  P-KPPSTVTIPTIDLGGGVFESTVARKSVVAKVKDAMEKFGFFQAINHGIPLDVMEKMKDGIRGFHEQDP
Crahi.0578s0006|Crahi.0578s0006.1   50  Y-KPSSELRIPSIDFGGGVFESPVTRESVVEKLKDAVERYGFFQSINHGFPLELMQRMRDGVRAFHDQDP
Crahi.0612s0012|Crahi.0612s0012.1   50  Y-KPSSEVRIPTIDFGGGMFESAVTREILVAKVKHAVENYGFFQSINHGFPLDVMQRMRDGVRAFHDQDP
Desop.0248s0927|Desop.0248s0927.1   52  P-RPTSLMTLPAIDLGGGVFESKAMRENVVNKFKDAAEKFGFFHAINHGIPLDLVEKMKDGIRGFHEQDP
Dharra.00051477                     1   --------------------------------------------CINHGFPLDLMQRMREGVHAFHDQDP
Distr.0010s35900.1                  53  L-KAPSTVMIPTIDLGGGVFESKTTRESVIAEIKDAVEIFGFFQAINHGIPLDVMEKMKNGTREFHEQDP
Dtenuisiliqua.0003266               50  Y-KPTSEVKIPSIDFGGRVFESPATRESLVAKVKDAVEKYGFFQSINHGFPLDLMQRMREGVRAFHDQDP
Dtenuisiliqua.00017018              50  Y-KPSSEVRIPSIDFGGGVFKSPVTRESVVAQVKDGAETYGFFQCINHGFPLDLMQRMRDGVRVFHDQDP
Eusyr.0020s0284.1                   52  P-KPSSTVTIPTIDLGGCMFESTVARESVISKVRDAMENFGFFQVINHGIPLDVMEKMKNGTRVFHEQDP
Eusyr.0020s0286.1                   52  P-KPSSTVMIPTIDLGGCMFESRVVRERIIAEVKDASEKYGFFQVINHGIPLDVLEKMKKGTRAFHEQDP
Eusyr.0020s0287.1                   52  P-KPSSTVMIPTIDLGGCMFESRVVRERIIAEVKDASEKYGFFQVINHGIPLDVLEKMKKGTRAFHEQDP
Hincana.00012582                    64  Y-KPSSEVRIPSIDFGGGVFESPVTRESVVAKLKDAVENYGFFQSINHGFPLDLMKRMRDGVRAFHDQDP
Hincana.00034316                    46  P-KPPSTLTIPTIDFGGGVFESTVTRKEVVEKVKDAMEKFGFFQAINHGIPLEVMEKMKDGIRGFHGQDP
Hincana.00039109                    71  Y-KPSSEVRIPSIDFGGGVFESPVTRESVVAKVKDAVERYGFFQSINHGFPLDLMQRMRDGVRAFHDQDP
```

```
Isati.0111s0004|Isati.0111s0004.1    52  P-KPSSVVRIPTIDFNGGVFDSTTTRESVVAELKDAMEKFGFFQAINHGVPLDVMDKMIDGIRGFHGQDS
Isati.0576s0034|Isati.0576s0034.1    52  P-KPSSTVRIPTIDFNGGVFDSTTTRESVVAELKDAVEKFGFFQAINHGVPLDVMEKMIDGIRGFHGQDS
Isati.0905s0036|Isati.0905s0036.1    52  PKKPPSTMRIPTIDFNGGVFDSTITRESVVAKLKDAAEKFGFFQAINHGIPRDVMDKMIDGTRAFHDQDS
Mamar.0031s0026|Mamar.0031s0026.1    46  HKLSSMMVMIPTIGLRGGVFDSTVERESVVAEVKDAMEKFGFFKAINHGVSLDMMEKIRNGIRGFHEQDQ
Msinaica.00026727                    52  P-KPSSTMRIPTIDFEGGVFDSKERRESVVAKVEEAMEKFGFFQAINHGIPLHVMEKMIAGVRGFHEQDP
Mspinosa.00020787                    52  P-KPSSTMRIPTIDFEGGVFDSKETRERVVAEVEEAMEKFGFFQAINHGIPLHVMEKMIAGVRGFHEQDP
Mspinosa.00027863                    46  Y-KPSSDVRIPSIDLGGGVFEAPATRESVVAKVKYAAENYGFFQCMNHGFPLDLMQRMREGVRAFHDQDP
Msuffruticosa.00012470               52  P-KPSSTMRIPTIDFEGGVFHSKETRERVVAKVEEAMEKFGFFQAINHGIPLHVMEKMIAGVRGFHEQDP
Msuffruticosa.00036638               46  P-KPPSTLTIPTIDLGGGVFESTAARKEAVEKVKDAMEKFGFFQAINHGIPLDVMNKMKDGIRGFHEQDP
Myper.0004s0500|Myper.0004s0500.1    52  PNKPPSTVSIPTIDFNGGVFDSTITRESVVAKLKDAAEKFGFFQAINHGIPRDVMDKMIDGTRGFHDQDP
Myper.0004s0502|Myper.0004s0502.1    68  PNKPPSTVSIPTIDFNGGVFDSTITRESVVAKLKDAAEKFGFFQAINHGIPRDVMDKMIDGTRGFHDQDP
Rsativus.108851740                   50  Y-KPPSEVRIPSIDFGGGVFESPVTRESVVAKVKNAAENYGFFQSINHGFPLDLMQRMREGVRSFHDQDP
Sialb.0020s0698.1                    50  Y-KPSSEVRIPSIDFGGRVFESPVTRESLVAKVKEAVENYGFFQSINHGFPLDLMQRMRDGVRAFHDQDP
Sialb.0099s0052.1                    50  Y-KPSSEVRIPSIDFGGRVFESPVTRESVVAKVKHAVENYGFFQSINHGFPLDLMQRMREGVRAFHDQDP
Sialb.1587s0010.1                    50  Y-KPSSEVRIPSIDFGGRVFESPVTRESLVAKVKEAVENYGFFQSINHGFPLDLMQRMRDGVRAFHDQDP
Stapi.4447s0001|Stapi.4447s0001.1    53  PQPSSMVMRIPTIDLKGCVFESTITRESVVAEVKHAMEKFGFFLAINHGIPLDVMEKMKDGIRGFHDLDP
Stapi.7214s0002|Stapi.7214s0002.1    52  --NRSQGLRVRIHD-----------RESVVAEVKHAMEKFGFFLAINHGIPLDVMEKMKDGIRGFHDLDP

Alyli.0054s0075|Alyli.0054s0075.1    121 EVRKMFYSRDKTKIVKYNSNADLYDSPAASWRDTLTTFMAPDVPRAEDLPEICREVMLEYSKEVMKLAEL
Alyli.0282s0030|Alyli.0282s0030.2    121 EVRKMFYSRDKTKTVKYNSNADLYDSPAASWRDTLITFMAPDVPRAEDLPEICREVMLEFSKEVLKLAEL
AT2G25450|AT2G25450.1                115 EVRKMFYTRDKTKKLKYHSNADLYESPAASWRDTLSCVMAPDVPKAQDLPEVCGEIMLEYSKEVMKLAEL
Bostr.26326s0118|Bostr.26326s0118.1  116 EVKKMFYSRDKTKKVKYNSNGDIYDSPAASWRDTLSCVMAPKLPKAEDLPEICGEIMLEYSKEVTKLAEL
Braju.04G167000.1                    115 EARKRFYSREKTKAIKYNSNSDLYDSPAASWRDTLSCFMFPDVPKTDDLPDICREIMLDYSKRVMMFGEL
Braju.04G167100.1                    115 EARKRFYSREKTKAIKYNSNSDLYDSPAASWRDTLSCFMFPDVPKTDDLPDICREIMLDYSKRVMMFGEL
Braju.04G167200.1                    115 EARKRFYSREKTKAIKYNSNSDLYDSPAASWRDTLSCFMFPDVPKTDDLPDICREIMLDYSKRMMMFGEL
Brara.D01848|Brara.D01848.1          115 EARKRFYSREKTKAIKYNSNSDLYDSPAASWRDTLSCFMFPDVPKTDDLPDICREIMLDYSKRVMMFGEL
Brara.D01849|Brara.D01849.1          115 EARKRFYSREKTKAIKYNSNSDLYDSPAASWRDTLSCFMFPDVPKTDDLPDICREIMLDYSKRMMMFGEL
Brara.K01198|Brara.K01198.1          112 EARKMFYSRDKTKKVKYNSNVDLYDSPAASWRDTLSLFLAPDVPKAEDLPEICGEIILEYSQGVMKLAEL
Brepanda.00021005                    119 QVRKTFYTRDVNKRVKYFSNPDLFESPAASWRDTFTCFMFPDVPNKQDLPEICGEVMLEYSKEVMKFAEL
Brepanda.00026522                    121 EAKKKFYSRDKTKKVKYNSNSDLYDSPAASWRDTLSLFLAPDVPKAEELPEICREIILEYSQGVMKLAEL
Caamp.0105s0129.1                    115 EERKMFYSREKTKAIKYNTNSDLYDAPAASWRDTLSCFMFPDVPKTEDLPEVCREIMLDYSKRVMMFGEL
Crahi.0578s0006|Crahi.0578s0006.1    119 QVRKTFYTRDINKRVKYFSNPDLFDSPAASWRDTFTCFMFPDVPNKQDLPEICGEVMLEYSKEVMKFAEL
Crahi.0612s0012|Crahi.0612s0012.1    119 QVRKTFYTRDMDKRVKYFSNPDLFDSPAASWRDTFTCFMFPDVPKKEELPAICGEIMLEYSKEVMKFAEL
Desop.0248s0927|Desop.0248s0927.1    121 EVRKMFYSRDKTKIVKYNSNADLYDSPAASWRDTLTTFMAPDVPRAEDLPEICREIMLEYSKEVMKLAEL
Dharra.00051477                      27  EVRKTFYTRDMTKRVKYFSNPDLFDSPAASWRDTFTCFMFPDVPKKEELPEICGEIMLDYSKEVMKFAEL
Distr.0010s35900.1                   122 EVRKTFYSRDKTKKVNCFTNADLYDSPAASWRDTLTCFMAPDVPKADDLPQIFGEIMLDYSERVMKLGEL
Dtenuisiliqua.0003266                119 QVRKKFYTRDMDKRVKYFSNPDLFDSPAASWRDTFTCFMFPDVPKMEELPEICGY-------------EL
Dtenuisiliqua.00017018               119 QVRKTFYTRDMNKRVKYFSNPDLFESPAASWRDTFTCFMFPDVPNKEELPEICGDVMLEYSKEVMKFAEL
Eusyr.0020s0284.1                    121 QVRKKFYSRDKTKKVKYMSNADLYDSPAASWRDTLNCFMAPHVPKTDDLPEICREILLEYSKRVIKLGEL
Eusyr.0020s0286.1                    121 EERKKFYSRDKTKKVKYMSNFDIYDSPAASWRDTLSCFMAPDVPKAEDIPEICRDIMFDYSKRVMKLGEL
Eusyr.0020s0287.1                    121 EERKKFYSRDKTKKVKYMSNFDIYDSPAASWRDTLSCFMAPDVPKAEDIPEICRDIMFDYSKRVMKLGEL
Hincana.00012582                     133 EVRKTFYTRDMDKRVKYFSNPDLFDSPAASWRDTLTCFMFPDVPKKEELPEICGEIMLEYSKEVMKFAEL
Hincana.00034316                     115 EARKTFYSREKTKAIKYNSNSDLYDSPAASWRDTLSCFMFPDVPKTDDLPDLCREIMLDYSKRVKMFGEL
Hincana.00039109                     140 QVRKTFYTRDMNKRVKYFSNPDLFDSPAASWRDTFTCFMFPDVPKKEDLPEICGEIMLEYSKEVMKFAEL
Isati.0111s0004|Isati.0111s0004.1    121 EARKRFYSRDKTKLVKYHSNSDLYDSPAASWRDTLTLFVAPDVPKAEDLPEICGEIILEYSKEVMKLGEL
Isati.0576s0034|Isati.0576s0034.1    121 EARKRFYSRDKTKQVKYNSNSDLYDSPAASWRDTLSLFLAPDVPKPEDLPEICGEIILEYSKEVMKLGEL
Isati.0905s0036|Isati.0905s0036.1    122 EARKKFYSRDKTQLVKYYSNADLFDSPAASWRDTLSLFVAPDVPKAEDLPEICGDIILEYSKEVMKLGEL
Mamar.0031s0026|Mamar.0031s0026.1    116 EVRKMFYTRDKTKKVKYNTNADLYDSPTASWRDTLSCAMAPDVPKAEDLPEICGEIMLEYTKEVMKLAEL
Msinaica.00026727                    121 EARKRFYSRDKTKMVKYNSNSDLYDSPAASWRDTLSLFLAPD-------------IILEYSQGVKKLAEL
Mspinosa.00020787                    121 EARK--------------------------------------------------REIILEYSQGVMKLAEL
Mspinosa.00027863                    115 QVRKTFYTRDMSKRVKYFSNPDLFESPAASWRDTFTCFMFPDVPKKEELPEICGEIMLEYSKEVMKFAEL
Msuffruticosa.00012470               121 EARKRFYSRDKTKMVKYNSNSDLYDSPAASWRDTLSLFLAPDVPKAEDLPEICGEIILEYSQGVMKLAEL
Msuffruticosa.00036638               115 EARKIFYSREKNKAVKYHSNYDLYDAPAASWRDTLTCFMFPD---------------------------L
Myper.0004s0500|Myper.0004s0500.1    122 EARKRFYSRDKTKLVKYSSNSDLYDSPAASWRDTLSLFVAPDVPKAEDLPEICGDIILEYSKEVMKLGEL
Myper.0004s0502|Myper.0004s0502.1    138 EARKRFYSRDKTKLVKYSSNSDLYDSPAASWRDTLSLFVAPDVPKAEDLPEICGDIILEYSKEVMKLGEL
Rsativus.108851740                   119 QVRKTFYTRDMNKRVKYFSNPDLFNSPAASWRDTFTCFMFPDVPNKQDLPEICGEVMLEYSKEVMKFAEL
Sialb.0020s0698.1                    119 LVRKTFYTRDMDKRVKYFSNPDLFDSPAASWRDTLTCFMFPDVPKKDDLPEICGEIMLEYSKEVMKFAEL
Sialb.0099s0052.1                    119 QVRKTFYTRDMNKRVKYFSNPDLFDSPAASWRDTFTCFMFPDVPDKQDLPEICGEIMLEYSKEVMKFAEL
Sialb.1587s0010.1                    119 QVRKTFYTRDMDKRVKYFSNPDLFDSPAASWRDTLTCFMFPEVPKKEDLPEIVGEIMLEYSKEVMKFAEL
Stapi.4447s0001|Stapi.4447s0001.1    123 EVRKMFYSRDKTKMVKYYSNYDLYDSPAASWRDTLSLFLAPDVPKVEDLPEICGEIILEYSKAVMKLGEV
Stapi.7214s0002|Stapi.7214s0002.1    110 EVRKMFYSRDKTKMVKYYSNYDLYDSPAASWRDTLSLFLAPDVPKVEDLPEICGEIILEYSKAVMKLGEV

Alyli.0054s0075|Alyli.0054s0075.1    191 LFELLSEALGLSPNHLKEMDCTEGLVMLGHCYPPCPEPDRTFGGAPHTDRSFLTI--LLQ-DHIEGFQVL
Alyli.0282s0030|Alyli.0282s0030.2    191 LFELLSEALGLSPNHLKEMDCTEGLVMLGHCYPPCPEPDRTFGGAPHTDRSFLTL--LLQ-DHIEGFQVL
AT2G25450|AT2G25450.1                185 MFEILSEALGLSPNHLKEMDCAKGLWMLCHCFPPCPEPNRTFGGAQHTDRSFLTI--LLN-DNNGGLQVL
Bostr.26326s0118|Bostr.26326s0118.1  186 MFELLSEALGLSPNHLKEMDCAKGLLLLSDCFPPCPEPNRTLGRIQHTDRSFLTI--LLH-DHIEGLQVL
Braju.04G167000.1                    185 IFELISESLGLKPNHLKKMDCAKGLLMLCHCYPPCPEPDLTLGATQHTDRSFITI--LLQ-DHIGGLQVL
Braju.04G167100.1                    185 IFELISESLGLNPNHLKEMDCAKGLLMLCHCYPPCPEPDLTLGATQHTDRSFITI--LLQ-DHIGGLQVL
Braju.04G167200.1                    185 IFELISESLGLNPNHLKEMDCAKGLLMLCHCYPPCPEPDRTLGATQHTDRSFITI--LLQ-DHIGGLQVL
Brara.D01848|Brara.D01848.1          185 IFELISESLGLKPNHLKKMDCAKGLLMLCHCYPPCPEPDLTLGATQHTDRSFITI--LLQ-DHIGGLQVL
```

```
Brara.D01849|Brara.D01849.1         185 IFELISESLGLNPNHLKEMDCAKGLLMLCHCYPPCPEPDRTLGATQHTDRSFITI--LLQ-DHIGGLQVL
Brara.K01198|Brara.K01198.1         182 IFELLSESLGLSRNHLKELDCAKGLLMLCHCYPPCPEPDLTLGATQHTDRSFVTI--LLQ-DHIGGLQVL
Brepanda.00021005                   189 VFEMLSEALGLSPNYLKDIGCTKGLCMLCHCYPPCPEPDRTLGAAQHTDRTFLTIGELLQ-DHVGGLQVV
Brepanda.00026522                   191 IFELLSEALGLSRNHLKELDCAEGLLMLCHCYPPCPEPDLTLGATQHTDRSFITI--LLQ-DHIGGLQVL
Caamp.0105s0129.1                   185 IFELLSEALGLNPNHLKEMDCAKGLLMLCHCYTPCPEPDRTLGATQHTDRSFLTI--LLQ-DHIGGLQVL
Crahi.0578s0006|Crahi.0578s0006.1   189 VFELLSEALGLSPNYLKDIGCTKGLCMLCHCYPPCPEPDRTLGAAQHTDRTFLTI--LLQ-DDIGGLQVV
Crahi.0612s0012|Crahi.0612s0012.1   189 VFELLSEALGLSPNHLKDIGCTKGLCMLCHCYPPCPEPDRTLGAAQHTDRTFLTI--LLQ-DHIGGLQAV
Desop.0248s0927|Desop.0248s0927.1   191 LFELLSEALGLSPNHLKEMDCTEGLVMLGHCYPPCPEPDRTFGGAPHTDRSFLTI--LLQ-DHIEGFQVL
Dharra.00051477                     97  IFELLSEALGLRPSYLKDIGCAKGLSMLCHCYPPCPEPDRTLGAAQHTDRTFLTI--LLQ-DHIGGLQAV
Distr.0010s35900.1                  192 IFELLSEALGLNPNHLKELGCAEGLLMLCHYFPPCPEPDRTLGGAPHTDRSFLTI--LIQ-DHIGGLQVL
Dtenuisiliqua.0003266               176 VFELLSEALGLSPNHLKEIGCTKGLCMLCHCYPPCPEPDRTLGAAQHTDRTFLTI--LLQ-DDIGGLQVT
Dtenuisiliqua.00017018              189 IFELLSEALGLSPNHLKDIGCAKGLSMLCHCYPSCPEPDRTLGAAQHTDRTFLTI--LLQ-DNIGGLQAV
Eusyr.0020s0284.1                   191 IFELLSEALGLSPNYLKEMDCAKGLLMLCHFFPPCPEPDLTLGGAQHTDRSFLTI--LLQ-DHIGGLQVL
Eusyr.0020s0286.1                   191 ISELLSEALGLNPNHLKEMDCTKGLWVLCHFFPPCPEPDLTLGASQHTDRSFLTI--LLQ-DHIGGLQVL
Eusyr.0020s0287.1                   191 ISELLSEALGLNPNHLKEMDCTKGLWVLCHFFPPCPEPDLTLGASQHTDRSFLTI--LLQ-DHIGGLQVL
Hincana.00012582                    203 VFELLSEALGLSPNHLKDIGCTKGLCMLCHCYPPCPEPDRTLGAAQHTDRTFLTI--LLQ-DHIGGLQVV
Hincana.00034316                    185 IFELISESLGLNPNHLKEMDCAKGLLMLCHCYPPCPEPDLTLGATQHTDRSFITI--LLQ-DHIGGLQVL
Hincana.00039109                    210 VFELLSEALGLGPNYLKDIGCTKGLCMLCHCYPPCPEPDRTLGAAQHTDRTFLTI--LLQ-DHIGGLQVV
Isati.0111s0004|Isati.0111s0004.1   191 IFELFSEALGLRPNHLKDMDCAKGLLMLCHCYPPCPEPDLTLGATQHTDRSFFTI--LLQ-DHIGGLQVV
Isati.0576s0034|Isati.0576s0034.1   191 IFELLSEALGLRPNHLKDMDCAKGLLMLCHCYPHCPEPDLTLGATQHTDRSFITI--LLQ-DHIGGLQVL
Isati.0905s0036|Isati.0905s0036.1   192 IFELFSEALGLSPNHLKEMDCAKGLWMLCHCYPPCPEPDLTLGAAQHTDRSFFTI--LLQ-DDIGGLQVV
Mamar.0031s0026|Mamar.0031s0026.1   186 MFELLSEALRLSPNHLKEMDCTKGLLLLCHCFPP------------------FTI--LLH-DHIGGLQVL
Msinaica.00026727                   178 IFELLSEALGLSSNHLKELDCAKGLLMLCHCYPPCPEPDLTLGATQHTDRSFITI--LLQ-DHIGGLQVL
Mspinosa.00020787                   142 IFELLSEALGLSSNHLKELDCAKGLLMLCHCYPPCPEPDLTLGATQHTDRSFITI--LLQ-DHIGGLQVL
Mspinosa.00027863                   185 IFELLSEALGLSPNHLKDIGCSKGLCMLCHCYPPCPEPDRTLGAAQHTDRTFLTI--LLQ-DHIGGLQAV
Msuffruticosa.00012470              191 IFELLSEALGLSSNHLKELDCAKGLLMLCHCYPPCPEPDLTLGATQHTDRSFITI--LLQ-DYIGGLQVL
Msuffruticosa.00036638              158 IFELLSESLGLNPNHLKEMDCAKGMLMLCHCYPPCPEPDLTLGATQHTDRSFFTI--LLQ-DHIGGLQVL
Myper.0004s0500|Myper.0004s0500.1   192 IFELFSEALGLSPNHLKEMDCAKGLWMLCHCYPPCPQPDLTLGAAQHTDRSFFTI--LLQ-DDIGGLQVV
Myper.0004s0502|Myper.0004s0502.1   208 IFELFSEALGLSPNHLKEMDCAKGLWMLCHCYPPCPQPDLTLGAAQHTDRSFFTI--LLQ-DDIGGLQVV
Rsativus.108851740                  189 VFELLSEALGLNHNHLKDIGCTKGLCMICHCYPPCPEPDRTLGAAQHTDRTFLTI--LLQ-DHIGGLQVL
Sialb.0020s0698.1                   189 VFELLSEALGLSPNHLKDIGCTKGFCMLCHCYPPCPEPDRTLGAAQHTDRTFLTI--LLQ-DDIGGLQAV
Sialb.0099s0052.1                   189 VFELLSEALGLSPNHLKDIGCTKGLCMLCHCYPPCPEPDRTLGAAQHTDRTFLTI--LLQ-DHIGGFQAV
Sialb.1587s0010.1                   189 VFELLSEALGLSPNHLKDIGCTKGFCMLCHCYPPCPEPDRTLGAAQHTDRTFLTI--LLQ-DDIGGLQAV
Stapi.4447s0001|Stapi.4447s0001.1   193 LFELISEALGLSPNHLKEMDCTKGLVMLGQCYPHCPEPDLTLGAAQHTDRSFLTV--LLPDDEIGGLQVL
Stapi.7214s0002|Stapi.7214s0002.1   180 LFELISEALGLSPNHLKEMDCTKGLVMLGQCYPHCPEPDLTLGAAQHTDRSFLTV--LLPDDEIGGLQVL

Alyli.0054s0075|Alyli.0054s0075.1   258 HEECWIDVPPNPKALIINIGDFLQ------------LLSNDKFVSAEHRILANSRDEPRISITCFIVH--
Alyli.0282s0030|Alyli.0282s0030.2   258 HEGCWIDVSPNPKALIINIGDFLQ------------LLSNDKFVSAEHRILANSRDEPRISIACFIVH--
AT2G25450|AT2G25450.1               252 YDGYWIDVPPNPEALIFNVGDFLQ------------LISNDKFVSMEHRILANGGEEPRISVACFFVHTF
Bostr.26326s0118|Bostr.26326s0118.1 253 HDGYWIDVTPDPEAFIINVGDLLQ------------LITNDKFVSVEHRILANRREEPRISVGCFFVH--
Braju.04G167000.1                   252 HDGYWIDVPPNPNALILNVGDLLQ------------LITNDKFVSVEHRVLANGGKEPRTSVASFFVH--
Braju.04G167100.1                   252 HDGYWIDVPPNPNALILNVGDLLQ------------LITNDKFVSVEHRVLANGGEEPRTSIASFFVH--
Braju.04G167200.1                   252 HDGYWIDVPPNPNALILNVGDLLQ------------LITNDKFVSVEHRVLANGGEEPRTSIASFFVH--
Brara.D01848|Brara.D01848.1         252 HDGYWIDVPPNPNALILNVGDLLQ----------------------------------------------
Brara.D01849|Brara.D01849.1         252 HDGYWIDVPPNPNALILNVGDLLQ------------------------------------ASCCLRI---
Brara.K01198|Brara.K01198.1         249 HDGYWIDVPPTPGALILNTGDLLQ------------LITNDKFVSVEHRVLANGRDEPRTSVASFFVH--
Brepanda.00021005                   258 HDGYYFDVPVNPDAIIVNIGDLLQ------------------------------------AISSFFIH--
Brepanda.00026522                   258 HDGYWIDVPPTPEALILN------------------LITNDKFVSVEHRVLANGRDEPRTSVASFFVH--
Caamp.0105s0129.1                   252 HDGYWIDVPPNPKALILNVGDLLQ------------LITNDKFVSVEHRVLANGGDQERISVASFFVH--
Crahi.0578s0006|Crahi.0578s0006.1   256 HDGYYFDVPVNPNAIIVNIGDLLQ------------LITNDKFISVEHRVMANRSNHPRIAVSSFFVH--
Crahi.0612s0012|Crahi.0612s0012.1   256 HDGYYIDVPVNPDAIIVNIGDLLQ------------LVTNDKFVSVEHRVMANGGKQPRIAVSSFFVH--
Desop.0248s0927|Desop.0248s0927.1   258 HEGCWIDVPPNPKALIINIGDFLQ------------LLSNDKFVSAEHRILANSRDEPRISITSFIVH--
Dharra.00051477                     164 HDGYYFDVPVNPDAII--------------------LITNDKFISVEHRVLANGSNHPRIAVSSFFVH--
Distr.0010s35900.1                  259 HNGYWIDVPPTLGAVVVNVGDLLQ------------LITNDKFVSVEHKVLATRGEEPRISIASFFAH--
Dtenuisiliqua.0003266               243 HDGYYFD-----------------------------LVTNDKFVSVEHRVMANRGNQPRIAVSSFFVH--
Dtenuisiliqua.00017018              256 HDGYYFD-----------------------------LITNDKFISVEHRVLANGSNQPRIAVSSFFVH--
Eusyr.0020s0284.1                   258 HDGYWIDVPPTPGALIVNVGDFLQ------------LMTNDKFVSVEHRVLATRGEEPRVSIACFFVH--
Eusyr.0020s0286.1                   258 NDGCWIDVPPTPGALIVNVGDYLQ------------LMTNDKFVSVEHRVLATRCDEPRVSIAAFFVH--
Eusyr.0020s0287.1                   258 NDGCWIDVPPTPGALIVNVGDYLQ------------LMTNDKFVSVEHRVLATRCDEPRVSIAAFFVH--
Hincana.00012582                    270 HDGYYIDVP---------------------------LVTNDKFVSVEHRVMANRGKQPRIAVSSFFVH--
Hincana.00034316                    252 HDGYWIDVPPNPNALILN------------------LITNDKFVSVEHRVLANGGKEPRTSIASFFVH--
Hincana.00039109                    277 HDGYYFDVPVDPNAIIVNIGDLLQASSYEIKRISNHLITNDKFISVEHRVLANGSNHPRIAVSSFFVH--
Isati.0111s0004|Isati.0111s0004.1   258 HDGYYIDVPPTEGALIINVGDLLQ------------LITNDKFVSVEHRVLANGSKEPRTSVASFFVH--
Isati.0576s0034|Isati.0576s0034.1   258 HDGYWIDVPPSQGALILNVGDLLQ------------LITNDKFVSVEHRVLANGSKEPRTSIASFFVH--
Isati.0905s0036|Isati.0905s0036.1   259 HDGYYIDVLPAEGALIINVGDLLQ------------LITNDKFVSVEHRVLANRTKELRTSISSFFVH--
Mamar.0031s0026|Mamar.0031s0026.1   235 HDESWIDVSPDPEALIFNVGDLLQ------------LMTNDKFVSVEHRVLANTGEEPRISVACFFVN--
Msinaica.00026727                   245 HDGYWIDVPPTPGALILNGGDLLQ--------------------------ATNGSNEPRTSVASFFVH--
Mspinosa.00020787                   209 HDGYWIDVPPTPGALILNGGDLLQ--------------------------ATNGSKEPRTSVASFFVH--
Mspinosa.00027863                   252 HDGYYFDVPVNPEAII--------------------LITNDKFISVEHRVLANGSNHPRIAVSSFFVH--
Msuffruticosa.00012470              258 HDGYWIDVPPTPGALILNGGDLLQASLLLSFFFLKRLITNDKFVSVEHRVLANGSNEPRTSVASFFVH--
```

```
Msuffruticosa.00036638            225 HDGYYIDVPPNPHALILN------------------LITNDKFVSVEHRVLANGGEEPRISIASFFVH--
Myper.0004s0500|Myper.0004s0500.1 259 HDGYYIDVLPAEGALIINVGDLLQ------------LITNDKFVSVEHRVLANGTKKLRTSISSFFVH--
Myper.0004s0502|Myper.0004s0502.1 275 HDGYYIDVLPAEGALIINVGDLLQ------------LITNDKFVSVEHRVLANGTKKLRTSISSFFVH--
Rsativus.108851740                256 HDGYYFDSPVNPDAIIVNIGDLLQ------------LITNDKFISVEHRVLANGSNHPRIAVSCFFVH--
Sialb.0020s0698.1                 256 QDGYYIDVPVNPDAIIVNIGDLLQ------------LVTNDKFVSVEHRVMANGSKQPRIAVSSFFVH--
Sialb.0099s0052.1                 256 HDGYYFDVPVNPDAIIVNIGDLLQ------------LITNDKFISVEHRVLANGSNHSRIAVSSFFVH--
Sialb.1587s0010.1                 256 HDGYYIDVPVNPDAIIVNIGDLLQ------------LVTNDKFVSVEHRVMANGSKQPRIAVSSFFVH--
Stapi.4447s0001|Stapi.4447s0001.1 261 HDGYYIDVPPTQGALTVNVGDLLQ------------LLSNDKFTSVEHRVLPTRSKEPRISVTTFFVH--
Stapi.7214s0002|Stapi.7214s0002.1 248 HDGYYIDVPPTQGALTVNVGDLLQ-----------------------------------ASCCSHLKS--

Alyli.0054s0075|Alyli.0054s0075.1 314 PLPSS-KVYGPIKELLSEQNPPKYRESTAETSNHYVPRK--------------LGGNSSLDHLRI
Alyli.0282s0030|Alyli.0282s0030.2 314 PLPSS-KVYGPIKELLSEQNPPKYRESTAETSNHYVPRK--------------LGGNSSLDHLRI
AT2G25450|AT2G25450.1             310 TSPSS-RVYGPIKELLSELNPPKYRDTTSESSNHYVARK--------------PNGNSSLDHLRI
Bostr.26326s0118|Bostr.26326s0118.1 309 TSPSS-RVYGPIKELLSEQNPPKYRDTTAGASNHYVARK--------------LNGNASLDHLRI
Braju.04G167000.1                 308 PPSTSPRVYGPIKELLSEENPPKYRETTPEASNHYVARK--------------RDGNNSLSHLRI
Braju.04G167100.1                 308 PPSTSPRVYGPIKELLSEENPPKYMETTSEASNHYVARK--------------RDGNNSLSHLRI
Braju.04G167200.1                 308 PPSTSPRVYGPIKELLSEENPPKYRETTPEASNHYVARK--------------RDGNNSLSHLRI
Brara.D01848|Brara.D01848.1       275 --------------------------------------------------------ASCCLHL--
Brara.D01849|Brara.D01849.1       282 -----------IKRSISKKTRSS------------------------------------------
Brara.K01198|Brara.K01198.1       305 PSSSS-RVYGPIKELLSEQNPPKYRDTTAEASNHYVPRK--------------GVGNASLSHLRI
Brepanda.00021005                 290 PPTSA-SVYGPIKELLSEENPPKYRETPAGYSNHFVPRQ--------------RH----------
Brepanda.00026522                 308 PTSS--RVYEPIKELLSEQNPPRYRGTTAEASNHYVPRN--------------GVGNSSLSHLRI
Caamp.0105s0129.1                 308 SPSTSSRVYGPIKELLSEQNPPKYRETTSEASNHYVARK--------------LDGNSSLSHLRI
Crahi.0578s0006|Crahi.0578s0006.1 312 PPTSP-SVYGPIKELLSEENPPKYRETPAGYSNHFVPRQ--------------RHGNNSLSHLRI
Crahi.0612s0012|Crahi.0612s0012.1 312 PPTSA-SVYGPIKELLSEENPPKYRETPAGYSNHFVPRE--------------RRGNNSLSHLRI
Desop.0248s0927|Desop.0248s0927.1 314 PLPSS-KVYGPIKELLSEQNPPKYRESTAETSNHYVPRK--------------LGGNSSLDHLRI
Dharra.00051477                   212 PPTSA-SVYGPIKELCSENNPPKYREAPAGYSNHFVPRQ--------------LHGNNSLSHLRI
Distr.0010s35900.1                315 PRPS--RIYGPIKELLSEQNPPKYRDTTTEASNHYVARI--------------PGGNASLQHLRI
Dtenuisiliqua.0003266             282 PPTSA-SVFGPIKELLSEENPPKYRETPASYSNHFVPRQ--------------RVGNNSLSHLRI
Dtenuisiliqua.00017018            295 PPTSA-SVVGPIKELLSENNPPKYRETPAGYSNHFVPRQ--------------RHGNNSLSHLRI
Eusyr.0020s0284.1                 314 HLSSQ-RVYGPIKELLSEQNPPKYRDTTPEASNHYVPRT--------------PGLNSSLLHLRI
Eusyr.0020s0286.1                 314 NLSS--RAYGPIKELLSEQNPPKYRETTPEVSNHYVPRETPPEVSNHCVPRTPGVNNTSLLHLRI
Eusyr.0020s0287.1                 314 NLSS--RAYGPIKELLSEQNPPKYRETTPEVSNHYVPRETPPEVSNHCVPRTPGVNNTSLRHLRI
Hincana.00012582                  311 PPTSA-SVFGPIKELLSEDNPPKYRETPAGYSNHFVPRQ--------------RHGNNSLSHLRI
Hincana.00034316                  302 PPSETPRVYGPIKELLSEENPPKYRETTSEASNHYVPRK--------------RDGNNSLSHLRI
Hincana.00039109                  345 PPTSA-SVYGPIKELLSEDNPPKYRETPAGYSNHFVPRQ--------------RHGNNSLSHLRI
Isati.0111s0004|Isati.0111s0004.1 314 PPSST-SVYGPIKELLSEENPPKYRETNAEASNHYVARK--------------RDGNSSLSHLRI
Isati.0576s0034|Isati.0576s0034.1 314 PPSST-SVYGPIKELLSEQNPAKYRETNAEASNHYVPRK--------------GGVNSSLSHLRI
Isati.0905s0036|Isati.0905s0036.1 315 PPSSS-TVYGPIKELLSEQNPPKYRETGAVASNHFVARK--------------SDGNSSLSHLRI
Mamar.0031s0026|Mamar.0031s0026.1 291 TTPSS-RVYGPIKELLSEQNPPKYKGTTAEASNHYVARK--------------LNVNASLDHLRI
Msinaica.00026727                 287 PSSS--RVYGPIKELLSEQNPPKYRETTAEASNHYVPRK--------------GVGNSSLSHLRI
Mspinosa.00020787                 251 PSSS--RVYGPIKELLSEQNPPKYRETTAEASNHYVPRK--------------GVGNSSLSHLRI
Mspinosa.00027863                 300 PPTST-SVYGPIKELLSENNPPKYREAPAGLSNHFVPRK--------------RHGNNSLSHLRI
Msuffruticosa.00012470            326 PSSS--RVYGPIKELLSEQNPPKYRETTAEASNHYVPRK--------------GVGNSSLSHLRI
Msuffruticosa.00036638            275 PPLASPRVYGPIKELLSEENPPKYRETTSEASNHYVARK--------------PSGNNSLSHLRI
Myper.0004s0500|Myper.0004s0500.1 315 PPSSS-TVYGPIKELLSEQNPPKYRETTSEASNHYVARK--------------RDGNNSLSHLRI
Myper.0004s0502|Myper.0004s0502.1 331 PPSSS-TVYGPIKELLSEQNPPKYRETTSEASNHYVARK--------------RDGNNSLSHLRI
Rsativus.108851740                312 PPTDA-SVYGPIKELLSEDNPPKYKETPAGFFNHFVPRQ--------------LHCNNSLSHLRI
Sialb.0020s0698.1                 312 PPTSA-SVYGPIKELLSEENPPKYRETPGGYSNHFVPRQ--------------RVGNNSLSHLRI
Sialb.0099s0052.1                 312 PPTSA-SVIGPIKELLSEDNPPKYRETPAGFSNHFVPRQ--------------RHGNNSLSHLRI
Sialb.1587s0010.1                 312 PPTSA-SVYGPIKELLSEENPPKYRETPAGYSNHFVPRQ--------------RVGNNSLSHLRI
Stapi.4447s0001|Stapi.4447s0001.1 317 PSPTS-RVYGPIKELLSEQNPPKYRDTTVDHSNHYVPRT--------------KVGNSSLSHLRI
Stapi.7214s0002|Stapi.7214s0002.1 280 ------------NELLSEQNPPKYRETTAEASNHYVPRK--------------GDGNSSLSHLRI
```

Supplementary Figure S5: GSL-OH clade alignment using MUSCLE. Highlighted in blue are the amino acids that differ at that position.

Supplementary Table S1. Phylogenetic relationship and glucosinolate chemotype of species used in this work.

The species and their lineage and tribe membership within the Brassicaceae are listed. Additionally the glucosinolate chemotype is provide with regards to if there are methionine derived glucosinolates (methoionine), Methylsulfinyl, But-3-enyl or 2HB3 glucosinolate detected within each species using the provided citations. Genes PAV shows if the data in the phylogenies show that GSL-OH or AOP indicates tha tthe gene is present or absent. HPLC and presence of stop codons or active site mutations was used to classify if a present GSL-OH was Functional or Non-Functional. AOP was not functionally validated and left as solely present/absent. NA means that there is no available information on the glucosinolates. In Lab, means that a sample of the species was obtained and tested in the laboratory.

	Classification		Glucosinolate Chemotype				Gene PAV		
**Species**	**Lineage (Supertribe)**	**Tribe**	**Methionine**	**MSO**	**but-3-enyl**	**2HB3**	**GSL-OH**	**AOP**	**Citation for source of glucosinolate phenotype described in previous columns**
Arabidopsis lyrata	I - Camelinodae	Arabidopsideae trib. nov.	Present	Present	Absent	Absent	Absent	Present	Clauss, M.J., Dietel, S., Schubert, G. *et al.* Glucosinolate and Trichome Defenses in a Natural *Arabidopsis lyrata* Population. *J Chem Ecol* **32**, 2351–2373 (2006). https://doi.org/10.1007/s10886-006-9150-8
Arabidopsis thaliana	I - Camelinodae	Arabidopsideae trib. nov.	Present	Present	Present	Present	Functional	Present	Windsor, A. J., Reichelt, M., Figuth, A., Svatoš, A., Kroymann, J., Kliebenstein, D. J., ... & Mitchell-Olds, T. (2005). Geographic and evolutionary diversification of glucosinolates among near relatives of Arabidopsis thaliana (Brassicaceae). *Phytochemistry*, *66* (11), 1321-1333.
Boechera stricta	I - Camelinodae	Boechereae	Present	Present	Absent	Absent	Non-functional	Absent	Fahey, J. W., 2001
Brassica juncea ssp. integrifolia MIZ-19 v1.1	II - Brassicodae	Brassiceae	Present	Present	Present	Present	Functional	Present	Bhandari, S. R., Jo, J. S., & Lee, J. G. (2015). Comparison of glucosinolate profiles in different tissues of nine Brassica crops. *Molecules*, *20* (9), 15827-15841.
Brassica oleracea capitata v1.0	II - Brassicodae	Brassiceae	Present	Present	Present	Present	Absent	Absent	Iwar, K., Desta, K. T., Ochar, K., & Kim, S. H. (2024). Unveiling Glucosinolate Diversity in Brassica Germplasm and In Silico Analysis for Determining Optimal Antioxidant Potential. *Antioxidants*, *13* (3), 376.
Brassica rapa	II - Brassicodae	Brassiceae	Present	Present	Present	Present	Functional	Present	Iwar, K., Desta, K. T., Ochar, K., & Kim, S. H. (2024). Unveiling Glucosinolate Diversity in Brassica Germplasm and In Silico Analysis for Determining Optimal Antioxidant Potential. *Antioxidants*, *13* (3), 376.
Brassica repanda	II - Brassicodae	Brassiceae	Present	Present	Absent	Absent	Non-functional	Present	Horn, P. J., & Vaughan, J. G. (1983). Seed glucosinolates of fourteen wild Brassica species. *Phytochemistry*, *22* (2), 465-470.
Cakile maritima v1.1	II - Brassicodae	Brassiceae	Present	Present	Present	Absent	Absent	Present	Fahey, J. W., 2001
Camelina sativa Prytzh v1.1	I - Camelinodae	Camelineae I	Present	Present	Absent	Absent	Absent	Present	Processed and analyzed samples in lab
Capsella grandiflora v1.1	I - Camelinodae	Camelineae I	Present	Present	Absent	Absent	Absent	Present	Czerniawski, P., Piasecka, A., & Bednarek, P. (2021). Evolutionary changes in the glucosinolate biosynthetic capacity in species representing Capsella, Camelina and Neslia genera. *Phytochemistry*, *181*, 112571.
Capsella rubella v1.1	I - Camelinodae	Camelineae I	Present	Present	Absent	Absent	Absent	Present	Czerniawski, P., Piasecka, A., & Bednarek, P. (2021). Evolutionary changes in the glucosinolate biosynthetic capacity in species representing Capsella, Camelina and Neslia genera. *Phytochemistry*, *181*, 112571.
Carrichtera annua	II - Brassicodae	Brassiceae	Present	Present	Present	Absent	Absent	Present	Daxenbichler, M. E., Spencer, G. F., Carlson, D. G., Rose, G. B., Brinker, A. M., & Powell, R. G. (1991). Glucosinolate composition of seeds from 297 species of wild plants. Phytochemistry, 30(8), 2623-2638.
Caulanthus amplexicaulis v1.1	II - Brassicodae	Thelypodieae	Present	Present	Present	Present	Functional	Absent	Relative does not have it; Daxenbichler, M. E., Spencer, G. F., Carlson, D. G., Rose, G. B., Brinker, A. M., & Powell, R. G. (1991). Glucosinolate composition of seeds from 297 species of wild plants. Phytochemistry, 30(8), 2623-2638.
Crambe hispanica v1.1	II - Brassicodae	Brassiceae	Present	Present	Present	Present	Functional	Present	Fahey, J. W., 2001
Descurainia pinnata	I - Camelinodae	Descurainieae	Present	Present	Present	Present	Functional	Present	Fahey, J. W., 2001
Descurainia sophioides v1.1	I - Camelinodae	Descurainieae	Present	Present	Present	Present	Functional	Present	Fahey, J. W., 2001
Diplotaxis acris	II - Brassicodae	Brassiceae	Present	Unknown	Unknown	Unknown	Absent	Absent	NA
Diplotaxis erucoides	II - Brassicodae	Brassiceae	Present	Present	Present	Absent	Absent	Present	D'Antuono, L. F., Elementi, S., & Neri, R. (2008). Glucosinolates in Diplotaxis and Eruca leaves: Diversity, taxonomic relations and applied aspects. *Phytochemistry*, *69* (1), 187-199.

Diplotaxis harra	II - Brassicodae	Brassiceae	Present	Present	Present	Absent	Non-functional	Present	D'Antuono, L. F., Elementi, S., & Neri, R. (2008). Glucosinolates in Diplotaxis and Eruca leaves: Diversity, taxonomic relations and applied aspects. *Phytochemistry* , *69* (1), 187-199.
Diplotaxis muralis	II - Brassicodae	Brassiceae	Present	Present	Absent	Absent	Absent	Absent	D'Antuono, L. F., Elementi, S., & Neri, R. (2008). Glucosinolates in Diplotaxis and Eruca leaves: Diversity, taxonomic relations and applied aspects. *Phytochemistry* , *69* (1), 187-199.
Diplotaxis tenuifolia	II - Brassicodae	Brassiceae	Present	Present	Absent	Absent	Absent	Absent	D'Antuono, L. F., Elementi, S., & Neri, R. (2008). Glucosinolates in Diplotaxis and Eruca leaves: Diversity, taxonomic relations and applied aspects. *Phytochemistry* , *69* (1), 187-199.
Diplotaxis tenuisiliqua	II - Brassicodae	Brassiceae	Present	Present	Present	Absent	Non-functional	Present	D'Antuono, L. F., Elementi, S., & Neri, R. (2008). Glucosinolates in Diplotaxis and Eruca leaves: Diversity, taxonomic relations and applied aspects. *Phytochemistry* , *69* (1), 187-199.
Diplotaxis viminea	II - Brassicodae	Brassiceae	Present	Absent	Absent	Absent	Absent	Absent	Daxenbichler, M. E., Spencer, G. F., Carlson, D. G., Rose, G. B., Brinker, A. M., & Powell, R. G. (1991). Glucosinolate composition of seeds from 297 species of wild plants. *Phytochemistry* , *30* (8), 2623-2638.
Diptychocarpus strictus v2.1	III - Hesperodae	Chorisporeae	Present	Unknown	Unknown	Unknown	Functional	Present	NA
Eruca sativa	II - Brassicodae	Brassiceae	Present	Present	Absent	Absent	Absent	Absent	Fahey, J. W., 2001; Bennett, R. N., Mellon, F. A., Botting, N. P., Eagles, J., Rosa, E. A., & Williamson, G. (2002). Identification of the major glucosinolate (4-mercaptobutyl glucosinolate) in leaves of Eruca sativa L.(salad rocket). Phytochemistry, 61(1), 25-30.
Eruca vesicaria v1.1	II - Brassicodae	Brassiceae	Present	Present	Absent	Absent	Absent	Absent	Fahey, J. W., 2001
Erysimum cheiranthoides	I - Camelinodae	Erysimeae	Present	Present	Absent	Absent	Absent	Absent	Fahey, J. W., 2001
Euclidium syriacum v1.1	III - Hesperodae	Euclidieae	Present	Present	Absent	Absent	Functional	Present	Brown, J., & Morra, M. J. (2005). *Glucosinolate-Containing Seed Meal as a Soil Amendment to Control Plant Pests: 2000-2002* (No. NREL/SR-510-35254). National Renewable Energy Lab.(NREL), Golden, CO (United States).
Eutrema salsugineum v1.0	II - Brassicodae	Eutremeae	Present	Present	Absent	Absent	Absent	Absent	aka Thellungiella halophila; Pang, Q., Chen, S., Li, L., & Yan, X. (2009). Characterization of glucosinolate—myrosinase system in developing salt cress Thellungiella halophila. *Physiologia Plantarum* , *136* (1), 1-9.
Hirschfeldia incana	II - Brassicodae	Brassiceae	Present	Present	Present	Present	Non-functional	Present	Fahey, J. W., 2001
Iberis amara v1.1	NA	Iberideae I	Present	Present	Present	Absent	Absent	Present	Fahey, J. W., 2001
Isatis tinctoria v1.1	II - Brassicodae	Isatideae	Present	Present	Present	Present	Functional	Present	Fahey, J. W., 2001
Lepidium sativum v1.1	I - Camelinodae	Lepidieae	Present	Present	Present	Present	Absent	Absent	Fahey, J. W., 2001
Lunaria annua v1.1	NA	Biscutelleae	Present	Present	Absent	Absent	Absent	Absent	Fahey, J. W., 2001
Malcolmia maritima v1.1	I - Camelinodae	Malcolmieae	Present	Present	Absent	Absent	Non-functional	Present (AOP3)	Fahey, J. W., 2001
Moricandia nitens	II - Brassicodae	Brassiceae	Present	Unknown	Unknown	Unknown	Absent	Present	Perfectti, F., Gómez, J. M., González-Megías, A., Abdelaziz, M., & Lorite, J. (2017). Molecular phylogeny and evolutionary history of Moricandia DC (Brassicaceae). *PeerJ* , *5* , e3964.; Daxenbichler
Moricandia sinaica	II - Brassicodae	Brassiceae	Present	Present	Present	Absent	Non-functional	Present	El-Mekkawy, S., Shahat, A. A., Alqahtani, A. S., Alsaid, M. S., Abdelfattah, M. A., Ullah, R., ... & Sobeh, M. (2020). A polyphenols-rich extract from moricandia sinaica boiss. exhibits analgesic, anti-inflammatory and antipyretic activities in vivo. *Molecules* , *25* (21), 5049.
Moricandia spinosa	II - Brassicodae	Brassiceae	Present	Unknown	Unknown	Unknown	Non-functional	Present	Perfectti, F., Gómez, J. M., González-Megías, A., Abdelaziz, M., & Lorite, J. (2017). Molecular phylogeny and evolutionary history of Moricandia DC (Brassicaceae). *PeerJ* , *5* , e3964.; Daxenbichler
Moricandia suffruticosa	II - Brassicodae	Brassiceae	Present	Unknown	Present	Unknown	Pending	Present	Perfectti, F., Gómez, J. M., González-Megías, A., Abdelaziz, M., & Lorite, J. (2017). Molecular phylogeny and evolutionary history of Moricandia DC (Brassicaceae). *PeerJ* , *5* , e3964.; Daxenbichler

Myagrum perfoliatum v2.1	II - Brassicodae	Isatideae	Present	Present	Present	Present	Functional	Present	H29; https://www.gcirc.org/publications/archives/irc-proceedings-until-2015/9th-irc-cambridge-united-kingdom-1995-vol-3-4
Raphanus sativus	II - Brassicodae	Brassiceae	Present	Present	Absent	Absent	Non-functional	Absent	In lab and Daxenbichler, M. E., Spencer, G. F., Carlson, D. G., Rose, G. B., Brinker, A. M., & Powell, R. G. (1991). Glucosinolate composition of seeds from 297 species of wild plants. *Phytochemistry* , *30* (8), 2623-2638.
Rorippa islandica v1.1	I - Camelinodae	Cardamineae	Present	Present	Present	Absent	Absent	Present	Fahey, J. W., 2001
Schrenkiella parvula v2.2	II - Brassicodae	Schrenkielleae trib. nov.	Present	Unknown	Unknown	Unknown	Absent	Present	NA
Sinapis alba v3.	II - Brassicodae	Brassiceae	Present	Present	Present	Present	Functional	Present	Fahey, J. W., 2001
Stanleya pinnata v1.1	II - Brassicodae	Thelypodieae	Present	Present	Present	Present	Functional	Present	Fahey, J. W., 2001
Thlaspi arvense v1.1	II - Brassicodae	Thlaspideae	Present	Present	Absent	Absent	Absent	Present	Fahey, J. W., 2001
**Species only included for Fig 1:**									
Brassica tournefortii	II - Brassicodae	Brassiceae	Present	Present	Present	Present	Present	Absent	Horn, P. J., & Vaughan, J. G. (1983). Seed glucosinolates of fourteen wild Brassica species. *Phytochemistry* , *22* (2), 465-470.
Brassica gravinae	II - Brassicodae	Brassiceae	Present	Present	Present	Present	Present	Absent	Horn, P. J., & Vaughan, J. G. (1983). Seed glucosinolates of fourteen wild Brassica species. *Phytochemistry* , *22* (2), 465-470.
Number of species with compound/enzyme (46 species)			46	39	23	14	13	31	
Number of species with compound/enzyme (50 species)	32		48	41	25	16	13	31	Moricandia nitens, spinosa, and suffruticosa can also be considered varieties of Moricandia arvensis. M. arvensis has been reported to make but-3-enyl and 2HB3; but it is unclear if the three include either.
				6					
Cleome albescens (outgroup)		Cleomaceae	Present	Absent	Absent	Absent	Absent	Absent	In lab

Supplementary Table S2. Genotype and glucosinolate information for species directly assayed for seed glucosinolates.

Shown are the Species directly assayed in this work for seed glucosinolates and the genotype information . The glucosinolate accumulation of GSL-OH related glucosinolates are shown in mAu per seed.

Species	GRIN ID	R2HB3	S2HB3	Allyl	But-3-Enyl	R:S Ratio
Descurainia pinnata	W653825			300	2365	
Descurainia pinnata	W660873	1835	78	1808	33	24:1
Descurainia pinnata	Not-GRIN; Phytozome	3961	802	1018	156	5:1
Descurainia sophia	W648991	38	246		847	1:6
Stanleya pinnata	W661078	31			3777	
Stanleya pinnata	W655938			4973	3727	
Stanleya pinnata	W659825	3307	2883		836	1.15:1
Isatis tinctoria	Not-GRIN; Phytozome	7231	6907		1574	1.05:1

Supplementary Table S3. AOP2 and AOP3 orthologues identified across all species tested.
Provided are the gene identities for those identified in each genome as an AOP2 or AOP3 orthologue in this work. NA means Supertribe not specified.

**Species queried**	**Lineage (Supertribe)**	**Genes identified as AOP2/3 orthologs**				
Arabidopsis lyrata	I - Camelinodae	AL6G49890				
Arabidopsis thaliana	I - Camelinodae	AT4G03060	AT4G03050			
Boechera stricta	I - Camelinodae					
Brassica juncea ssp. integrifolia MIZ-19 v1.1	II - Brassicodae	Braju.11G394800	Braju.02G245200	Braju.03G298800	Braju.13G336300	Braju.09G021200
Brassica oleracea capitata v1.0	II - Brassicodae					
Brassica rapa	II - Brassicodae	Brara.B02636	Brara.C02776	Brara.I00199		
Brassica repanda	II - Brassicodae	Brepanda.00039963				
Cakile maritima v1.1	II - Brassicodae	Camar.0095s0059				
Camelina sativa Prytzh v1.1	I - Camelinodae	CsPrytzh.02G028800				
Capsella grandiflora v1.1	I - Camelinodae	Cagra.2552s0023				
Capsella rubella v1.1	I - Camelinodae	Carub.0006s3401				
Carrichtera annua	II - Brassicodae	Cannua.00007407	Cannua.00007773			
Caulanthus amplexicaulis v1.1	II - Brassicodae					
Cleome albescens	I - Camelinodae					
Crambe hispanica v1.1	II - Brassicodae	Crahi.0108s0065	Crahi.1585s0003			
Descurainia pinnata	I - Camelinodae	Alyli.0009s0329				
Descurainia sophioides v1.1	I - Camelinodae	Desop.0039s0116				
Diplotaxis acris	II - Brassicodae					
Diplotaxis erucoides	II - Brassicodae	Derucoides.00034966				
Diplotaxis harra	II - Brassicodae	Dharra.00049988				
Diplotaxis muralis	II - Brassicodae					
Diplotaxis tenuifolia	II - Brassicodae					
Diplotaxis tenuisiliqua	II - Brassicodae	Dtenuisiliqua.0002623				
Diplotaxis viminea	II - Brassicodae					
Diptychocarpus strictus v2.1	III - Hesperodae	Distr.0010s95400				
Eruca sativa	II - Brassicodae					
Eruca vesicaria v1.1	II - Brassicodae					
Erysimum cheiranthoides	I - Camelinodae					
Euclidium syriacum v1.1	III - Hesperodae	Eusyr.0043s0042				
Eutrema salsugineum v1.0	II - Brassicodae					
Hirschfeldia incana	II - Brassicodae	Hincana00013037	Hincana00007386			
Iberis amara v1.1	NA	Ibeam.2784s0001	Ibeam.4665s0003	Ibeam.4043s0003		
Isatis tinctoria v1.1	II - Brassicodae	Isati.3575s0002	Isati.0596s0007	Isati.4898s0001		
Lepidium sativum v1.1	I					
Malcolmia maritima v1.1	NA	Mamar.0100s0003	Mamar.0100s0005			
Moricandia nitens	I - Camelinodae	Mnitens.00017464	Mnitens.00017074			
Moricandia sinaica	II - Brassicodae	Msinaica.00034235	Msinaica.00027664			
Moricandia spinosa	II - Brassicodae	Mspinosa.00010699	Mspinosa.00038965	Mspinosa.00055249	Mspinosa.00045033	
Moricandia suffruticosa	II - Brassicodae	Msuffruticosa.000092	Msuffruticosa.000231			
Myagrum perfoliatum v2.1	II - Brassicodae	Myper.0011s0541				
Raphanus sativus	II - Brassicodae					
Rorippa islandica v1.1	II - Brassicodae	Roisl.0082s0363				
Schrenkiella parvula v2.2	I - Camelinodae	Sp6g03090				
Sinapis alba v3.	II - Brassicodae	Sialb.0016s0175	Sialb.1049s0032	Stapi.0234s0018		
Stanleya pinnata v1.1	II - Brassicodae	Stapi.3195s0002				
Thlaspi arvense v1.1	II - Brassicodae					

Supplementary Table S4. Glucosinolate accumulation in individual plants for each GSL-OH gene being tested for function.

The table provided the glucosinolate accumulation of all GSL-OH related glucosinolates (R2HB, S2HB, Allyl and But-3-enyl) along with derived ratios obtained as described in each header. These are individual runs for each independent transgene for each gene as labeled in the first two columns. All units are in mAu per sample as the comparison is between compounds.

Gene	Transgene	R2HB	S2HB	allyl	But-3-enyl	2HB3 Sum = R2HB + S2HB	4C Alkenyl = 2HB3 + But-3-enyl	GSL-OH efficiency = 2HB3/4C Alkenyl	Enantiomeric = R2HB/2HB3
Brara.K01198	39	41	0	128	497	41	538	0.08	1.00
Brara.K01198	39	218	0	224	888	218	1106	0.20	1.00
Brara.K01198	39	368	0	371	1504	368	1872	0.20	1.00
Brara.K01198	39	158	0	177	739	158	897	0.18	1.00
Brara.K01198	39	65	0	72	215	65	280	0.23	1.00
Brara.K01198	40	456	0	205	493	456	949	0.48	1.00
Brara.K01198	40	489	0	180	321	489	810	0.60	1.00
Brara.K01198	40	616	0	223	212	616	828	0.74	1.00
Brara.K01198	40	99	0	170	888	99	987	0.10	1.00
Brara.K01198	45	530	8	402	1495	538	2033	0.26	0.99
Brara.K01198	45	426	0	245	972	426	1398	0.30	1.00
Brara.K01198	45	90	0	261	1367	90	1457	0.06	1.00
Brara.K01198	46	117	0	150	803	117	920	0.13	1.00
Brara.K01198	46	428	0	193	395	428	823	0.52	1.00
Brara.K01198	46	922	64	385	600	986	1586	0.62	0.94
Brara.K01198	39	632	0	482	1687	632	2319	0.27	1.00
Brara.K01198	39	1178	19	804	1853	1197	3050	0.39	0.98
Brara.K01198	39	778	0	751	2569	778	3346	0.23	1.00
Brara.K01198	39	575	0	714	3010	575	3585	0.16	1.00
Brara.K01198	40	1139	0	750	2156	1139	3295	0.35	1.00
Brara.K01198	40	853	0	423	834	853	1687	0.51	1.00
Brara.K01198	40	138	0	53	58	138	195	0.71	1.00
Brara.K01198	40	84	0	274	1210	84	1294	0.06	1.00
Brara.K01198	40	348	0	687	2551	348	2900	0.12	1.00
Brara.K01198	40	676	0	646	2445	676	3120	0.22	1.00
Brara.K01198	40	1868	25	786	698	1894	2591	0.73	0.99
Brara.K01198	45	774	0	555	1879	774	2653	0.29	1.00
Brara.K01198	45	186	0	611	2647	186	2833	0.07	1.00
Brara.K01198	45	263	0	537	2261	263	2524	0.10	1.00
Brara.K01198	45	1252	0	622	1270	1252	2521	0.50	1.00
Brara.K01198	45	254	0	601	2700	254	2953	0.09	1.00
Brara.K01198	45	995	0	250	327	995	1322	0.75	1.00
Brara.K01198	46	428	0	495	1982	428	2410	0.18	1.00
Brara.K01198	46	256	0	533	2146	256	2403	0.11	1.00
Brara.K01198	46	871	0	448	1131	871	2001	0.44	1.00
Brara.K01198	46	1293	20	491	558	1313	1871	0.70	0.98
Alyli.0282s0030	30f	462	102	314	124	564	688	0.82	0.82
Alyli.0282s0030	30f	178	38	1119	2931	216	3147	0.07	0.82
Alyli.0282s0030	30f	1445	330	1181	479	1775	2254	0.79	0.81
Alyli.0282s0030	30f	531	124	372	124	655	779	0.84	0.81
Alyli.0282s0030	30f	168	40	183	184	208	392	0.53	0.81
Alyli.0282s0030	30f	354	84	215	55	438	493	0.89	0.81
Alyli.0282s0030	30f	1133	254	836	200	1387	1587	0.87	0.82
Alyli.0282s0030	30f	41	0	145	396	41	437	0.09	1.00
Alyli.0282s0030	30f	63	14	367	1394	77	1471	0.05	0.82
Alyli.0282s0030	30f	429	93	559	695	522	1217	0.43	0.82
Alyli.0282s0030	30f	74	16	252	518	90	608	0.15	0.82
Alyli.0282s0030	30f	133	23	767	2148	156	2304	0.07	0.85
Alyli.0282s0030	30f	159	32	1403	3890	191	4081	0.05	0.83
Alyli.0282s0030	30f	87	18	472	1089	105	1194	0.09	0.83
Alyli.0282s0030	30f	199	44	130	58	243	301	0.81	0.82
Alyli.0282s0030	30f	974	216	792	234	1190	1424	0.84	0.82
Alyli.0282s0030	30f	546	118	438	167	664	831	0.80	0.82
Alyli.0282s0030	30f	867	198	598	197	1065	1262	0.84	0.81
Alyli.0282s0030	29g	512	112	401	210	624	834	0.75	0.82
Alyli.0282s0030	29g	1027	226	774	170	1253	1423	0.88	0.82
Alyli.0282s0030	29g	1008	220	682	101	1228	1329	0.92	0.82

Alyli.0282s0030	29g	1013	231	665	107	1244	1351	0.92	0.81
Alyli.0282s0030	29g	1640	373	1173	191	2013	2204	0.91	0.81
Alyli.0282s0030	29g	952	200	663	163	1152	1315	0.88	0.83
Alyli.0282s0030	29g	1167	243	946	188	1410	1598	0.88	0.83
Alyli.0282s0030	29g	320	71	246	113	391	504	0.78	0.82
Alyli.0282s0030	29g	427	91	326	159	518	677	0.77	0.82
Alyli.0282s0030	29g	472	109	348	72	581	653	0.89	0.81
Alyli.0282s0030	29g	974	196	697	144	1170	1314	0.89	0.83
Alyli.0282s0030	29g	1108	233	803	101	1341	1442	0.93	0.83
Alyli.0282s0030	29g	1884	361	1674	799	2245	3044	0.74	0.84
Alyli.0282s0030	29g	533	96	446	245	629	874	0.72	0.85
Alyli.0054s0075	15c	141	660	337	417	801	1218	0.66	0.18
Alyli.0054s0075	15c	27	115	311	1351	142	1493	0.10	0.19
Alyli.0054s0075	15c	142	694	384	429	836	1265	0.66	0.17
Alyli.0054s0075	15c	115	570	217	62	685	747	0.92	0.17
Alyli.0054s0075	15c	41	181	484	2440	222	2662	0.08	0.18
Alyli.0054s0075	15c	245	1238	444	121	1483	1604	0.92	0.17
Alyli.0054s0075	15c	121	623	257	127	744	871	0.85	0.16
Alyli.0054s0075	15c	126	703	203	17	829	846	0.98	0.15
Alyli.0054s0075	15c	197	962	423	136	1159	1295	0.89	0.17
Alyli.0054s0075	15c	30	148	54	23	178	201	0.89	0.17
Alyli.0054s0075	15c	44	232	94	39	276	315	0.88	0.16
Alyli.0054s0075	15c	93	423	242	63	516	579	0.89	0.18
Alyli.0054s0075	15c	186	884	410	89	1070	1159	0.92	0.17
Alyli.0054s0075	15c	70	342	162	72	412	484	0.85	0.17
Alyli.0054s0075	15c	44	204	598	2200	248	2448	0.10	0.18
Alyli.0054s0075	15c	28	123	170	456	151	607	0.25	0.19
Alyli.0054s0075	37b	45	232	1062	2448	277	2725	0.10	0.16
Alyli.0054s0075	37b	197	1008	900	491	1205	1696	0.71	0.16
Alyli.0054s0075	37b	144	735	670	230	879	1109	0.79	0.16
Alyli.0054s0075	37b	235	1186	1112	515	1421	1936	0.73	0.17
Alyli.0054s0075	37b	32	151	1031	2853	183	3036	0.06	0.17
Alyli.0054s0075	37b	184	956	740	276	1140	1416	0.81	0.16
Alyli.0054s0075	37b	198	1032	889	523	1230	1753	0.70	0.16
Alyli.0054s0075	37b	1283	308	911	226	1591	1817	0.88	0.81
Alyli.0054s0075	37b	252	1327	1154	511	1579	2090	0.76	0.16
Alyli.0054s0075	37b	263	1328	1204	680	1591	2271	0.70	0.17
Alyli.0054s0075	37b	22	108	676	1500	130	1630	0.08	0.17
Alyli.0054s0075	37b	43	225	581	1091	268	1359	0.20	0.16
Alyli.0054s0075	37b	32	172	895	1885	204	2089	0.10	0.16
Alyli.0054s0075	37b	94	496	394	165	590	755	0.78	0.16
Alyli.0054s0075	37b	139	641	577	397	779	1176	0.66	0.18
Alyli.0054s0075	37b	138	733	529	219	870	1090	0.80	0.16
Alyli.0054s0075	37b	34	168	727	1488	202	1690	0.12	0.17
Alyli.0054s0075	37b	24	123	705	1829	147	1976	0.07	0.16
Alyli.0054s0075	37b	177	939	656	243	1116	1359	0.82	0.16
AT2G25450	4d	57	152	269	1035	209	1243	0.17	0.27
AT2G25450	4d	15	33	234	1473	48	1520	0.03	0.31
AT2G25450	4d	16	38	301	1604	53	1657	0.03	0.29
AT2G25450	4d	50	141	344	1431	191	1622	0.12	0.26
AT2G25450	4d	78	222	511	2055	300	2355	0.13	0.26
AT2G25450	4d	15	34	253	1224	49	1273	0.04	0.30
AT2G25450	4d	14	48	207	1215	63	1277	0.05	0.23
AT2G25450	4d	50	148	289	1054	199	1253	0.16	0.25
AT2G25450	4d	38	106	282	1281	144	1425	0.10	0.26
AT2G25450	4d	20	69	161	1047	89	1136	0.08	0.23
AT2G25450	4d	26	78	202	852	104	956	0.11	0.25
AT2G25450	4d	29	76	320	1501	105	1606	0.07	0.28
AT2G25450	4d	24	64	291	1422	88	1510	0.06	0.27
AT2G25450	4d	41	113	196	822	154	976	0.16	0.27
AT2G25450	4d	0	15	61	252	15	267	0.06	0.00
AT2G25450	4d	42	116	260	1123	158	1281	0.12	0.27
AT2G25450	4d	19	50	183	1327	69	1396	0.05	0.27
AT2G25450	4d	45	126	253	1178	171	1349	0.13	0.26
AT2G25450	4b	18	48	340	1455	66	1521	0.04	0.27

AT2G25450	4b	15	41	296	1150	56	1205	0.05	0.27
AT2G25450	4b	23	70	79	259	93	352	0.26	0.25
AT2G25450	4b	38	100	256	1569	138	1707	0.08	0.28
AT2G25450	4b	31	90	204	585	121	707	0.17	0.26
AT2G25450	4b	21	60	274	1157	82	1238	0.07	0.26
AT2G25450	4b	19	59	303	1228	79	1307	0.06	0.25
AT2G25450	4b	22	52	327	1324	74	1397	0.05	0.29
AT2G25450	4b	19	50	319	1590	69	1659	0.04	0.27
AT2G25450	4b	39	117	291	1050	156	1206	0.13	0.25
AT2G25450	4b	13	36	216	1124	49	1173	0.04	0.27
AT2G25450	4b	37	99	345	1763	136	1899	0.07	0.27
AT2G25450	4b	29	79	236	1013	109	1121	0.10	0.27
AT2G25450	4b	0	27	160	677	27	704	0.04	0.00
AT2G25450	4b	22	72	315	1239	94	1334	0.07	0.24
AT2G25450	4b	35	101	312	2348	136	2484	0.05	0.26
AT2G25450	4b	62	170	454	2476	232	2708	0.09	0.27
AT2G25450	4b	35	97	262	1148	133	1281	0.10	0.26
Isati.0905s0036		135	503	498	1070	638	1708	0.37	0.21
Isati.0905s0036		29	113	271	996	142	1138	0.12	0.20
Isati.0905s0036		129	447	560	1412	576	1988	0.29	0.22
Isati.0905s0036		61	258	286	641	319	960	0.33	0.19
Stapi.7214s0002		0	242	259	639	242	881	0.27	0.00
Stapi.7214s0002		34	311	273	749	345	1094	0.32	0.10
Stapi.7214s0002		111	392	414	997	503	1500	0.34	0.22
Stapi.7214s0002		50	382	356	625	432	1057	0.41	0.12
Isati.0576s0034.1		283	67	449	1165	350	1515	0.23	0.81
Isati.0576s0034.1		753	201	242	40	954	994	0.96	0.79
Isati.0111s0004.1		154	0	393	1249	154	1403	0.11	1.00
Isati.0111s0004.3		258	0	154	228	258	486	0.53	1.00
Desop.0248s0927		114	475	338	788	589	1377	0.43	0.19
Desop.0248s0927		149	677	180	120	826	946	0.87	0.18
Desop.0248s0927		19	0	459	1890	19	1909	0.01	1.00
Isati.0111s0004.1		122	0	166	338	122	460	0.27	1.00
Isati.0111s0004.1		545	0	263	395	545	940	0.58	1.00
Myper.0004s0500		91	798	153	56	889	945	0.94	0.10
Myper.0004s0500		70	295	258	542	365	907	0.40	0.19
Myper.0004s0500		52	435	127	99	487	586	0.83	0.11
Myper.0004s0500		57	351	423	1099	408	1507	0.27	0.14
Myper.0004s0500		0	157	120	173	157	330	0.48	0.00
Crahi.0578s0006		0	82	47	48	82	130	0.63	0.00
Crahi.0578s0006		0	386	220	343	386	729	0.53	0.00
Crahi.0578s0006		0	346	131	136	346	482	0.72	0.00
Crahi.0578s0006		30	449		53	479	532	0.90	0.06
Stapi.4447s0001		0	98	145	317	98	415	0.24	0.00
Stapi.4447s0001		39	276	169	214	315	529	0.60	0.12
Stapi.4447s0001		35	301	167	197	336	533	0.63	0.10
Stapi.4447s0001		61	669		35	730	765	0.95	0.08
Isati.0905s0036		82	347	367	537	429	966	0.44	0.19
Isati.0905s0036		0	35	169	319	35	354	0.10	0.00
Isati.0905s0036		63	246	319	719	309	1028	0.30	0.20

Alyli.0282s0030	184	45	133	25	229	254	0.90	0.80
Alyli.0282s0030	214	50	159	25	264	289	0.91	0.81
Alyli.0282s0030	362	85	224	320	447	767	0.58	0.81
Myper.0004s0502	305	649	364	503	954	1457	0.65	0.32
Myper.0004s0502	48	111	80	75	159	234	0.68	0.30
Myper.0004s0502	37	63	143	246	100	346	0.29	0.37
Myper.0004s0502	272	590	317	369	862	1231	0.70	0.32
Braju.03G405000	140	0	439	1472	140	1612	0.09	1.00
Braju.03G405000	690	11	762	2183	701	2884	0.24	0.98
Braju.03G405000	343	0	457	1234	343	1577	0.22	1.00
Braju.03G405000	24	0	110	104	24	128	0.19	1.00
Braju.03G405000	445	0	357	981	445	1426	0.31	1.00
Braju.03G405000	291	0	824	2629	291	2920	0.10	1.00
Braju.03G405000	1238	14	840	1706	1252	2958	0.42	0.99
Braju.03G405000	1353	17	703	1391	1370	2761	0.50	0.99
Braju.03G405000	1956	23	753	526	1979	2505	0.79	0.99
Caamp.0105s0129	0	0	259	790	0	790	0.00	
Caamp.0105s0129	0	15	724	2953	15	2968	0.01	0.00
Caamp.0105s0129	0	16	569	2672	16	2688	0.01	0.00
Caamp.0105s0129	0	0	28	52	0	52	0.00	
Caamp.0105s0129	0	0	65	232	0	232	0.00	
Caamp.0105s0129	0	0	588	2534	0	2534	0.00	
Caamp.0105s0129	0	0	389	1743	0	1743	0.00	
Caamp.0105s0129	0	0	34	52	0	52	0.00	
Caamp.0105s0129	0	0	719	2916	0	2916	0.00	
Sialb.1587s0010	0	21	557	2180	21	2201	0.01	0.00
Sialb.1587s0010	0	50	546	2032	50	2082	0.02	0.00
Sialb.1587s0010	0	220	871	2658	220	2878	0.08	0.00
Sialb.1587s0010	0	55	247	757	55	812	0.07	0.00
Sialb.1587s0010	0	281	657	2714	281	2995	0.09	0.00
Sialb.1587s0010	402	1801	644	1221	2203	3424	0.64	0.18
Sialb.1587s0010	0	77	462	1962	77	2039	0.04	0.00
Eusyr0020s0286	193	43	334	1103	236	1339	0.18	0.82
Eusyr0020s0286	11	0	100	462	11	473	0.02	1.00
Eusyr0020s0286	143	29	327	1090	172	1262	0.14	0.83
Eusyr0020s0286	71	14	270	801	85	886	0.10	0.84
Braju04G167100	19	0	125	493	19	512	0.04	1.00
Braju04G167100	0	0	30	56		56	0.00	
Braju04G167200	113	0	83	224	113	337	0.34	1.00
Braju04G167200	25	0	17	25	25	50	0.50	1.00
Braju04G167200	34	0	52	71	34	105	0.32	1.00
Braju04G167200	125	0	123	200	125	325	0.38	1.00
Braju04G167200	398	43	140	125	441	566	0.78	0.90
Eusyr0020s0284	29	0	36	64	29	93	0.31	1.00
Eusyr0020s0284	19	0	184	836	19	855	0.02	1.00
Eusyr0020s0284	13	0	55	99	13	112	0.12	1.00
Eusyr0020s0287	533	139	187	130	672	802	0.84	0.79
Eusyr0020s0287	228	61	324	701	289	990	0.29	0.79

Supplementary Table S5. Active Site information for each GSL-OH gene identified in this study.

Shown is each the amino acids at the conserved 2ODD active site for each gene. The conserved active site AA is shown in row five as the header for each column. The Subclade for each GSL-OH gene is included for reference. The length of the predicted protein is also provided.

		Conserved 2ODD Active Site Amino Acids										
GSL-OH Subclade	Gene	T	S	L	Y	H	D	H	R	F	V	Protein Length (AAs)
A	AT2G25450	T	S	L	F	H	D	H	R	F	V	359
A	Alyli.0054s0075	T	T	L	Y	H	D	H	R	F	V	363
A	Alyli.0282s0030	T	I	L	Y	H	D	H	R	F	V	363
A	Bostr.26326s0118	T	S	L	F	H	D	H	R	F	V	358
A	Desop.0248s0927	T	T	L	Y	H	D	H	R	F	V	363
A	Mamar.0031s0026	T	S	L	F	DEL	DEL	H	R	F	T	340
B	Braju.04G167000	T	S	L	Y	H	D	H	R	R	V	358
B	Braju.04G167100	T	S	L	Y	H	D	H	R	R	V	358
B	Braju.04G167200	T	S	L	Y	H	D	H	R	F	V	358
B	Brara.D01848	T	S	L	Y	H	D	DEL	DEL	DEL	DEL	282
B	Brara.D01849	T	S	L	Y	H	D	I	K	DEL	DEL	283
B	Caamp.0105s0129	T	S	L	Y	H	D	H	R	F	S	358
B	Hincana00034316	T	S	L	Y	H	D	H	R	F	P	352
B	Msuffruticosa.00036638	T	T	L	Y	H	D	H	R	F	P	325
B'	Brepanda.00026522	T	S	L	Y	H	D	H	R	F	V	356
B'	Isati.0111s0004	T	T	L	Y	H	D	H	R	F	V	363
B'	Isati.0576s0034	T	S	L	Y	H	D	H	R	F	V	363
B'	Isati.0905s0036.1	T	S	L	Y	H	D	H	R	F	V	364
B'	Msinaica.00026727	T	S	L	Y	H	D	DEL	R	F	V	335
B'	Mspinosa.00020787	DEL	DEL	L	Y	H	D	DEL	R	F	V	299
B'	Msuffruticosa.00012470	T	S	L	Y	H	D	H	R	F	V	374
B'	Myper.0004s0500	T	S	L	Y	H	D	H	R	F	V	364
B'	Myper.0004s0502	T	S	L	Y	H	D	H	R	F	V	380
B'	Stapi.4447s0001	T	S	L	Y	H	D	T	V	DEL	P	366

B'	Stapi.7214s0002	T	S	L	Y	H	D	S	E	DEL	N	319
B'	Brara.K01198	T	S	L	Y	H	D	H	R	F	V	354
C	Brepanda.00021005	T	T	L	Y	H	D	DEL	DEL	I	T	329
C	Crahi.0578s0006	T	T	L	Y	H	D	H	R	V	T	361
C	Crahi.0612s0012	T	T	L	Y	H	D	H	R	V	T	361
C	Dharra.00051477	Protein not included as 2/3rds of 2ODD size										251
C	Dtenuisiliqua.00017018	T	T	L	Y	H	D	H	R	V	T	344
C	Dtenuisiliqua.00032667	T	T	L	Y	H	D	DEL	R	V	T	331
C	H.incana00012582	T	T	L	Y	H	D	DEL	R	V	T	360
C	Hincana00039109	T	T	L	Y	H	D	H	R	V	T	394
C	Mspinosa.00027863	T	T	L	Y	H	D	H	R	V	T	349
C	Rsativus.108851740	T	T	L	Y	H	D	H	R	V	T	361
C	Sialb.0020s0698	T	T	L	Y	H	D	H	R	V	T	361
C	Sialb.0099s0052	T	T	L	Y	H	D	H	R	V	T	361
C	Sialb.1587s0010	T	T	L	Y	H	D	H	R	V	T	361
	Distr.0010s35900	T	T	L	F	H	D	R	L	DEL	P	363
	Eusyr.0020s0284	T	N	L	F	H	D	S	L	DEL	P	363
	Eusyr.0020s0286	T	S	L	F	H	D	L	L	DEL	P	376
	Eusyr.0020s0287	T	S	L	F	H	D	L	L	DEL	P	376

Supplementary Table S6 - Primers used to amplify each gene for DNA and RNA testing.

Gene name	F	R
AL4G25790	ACAAGTTTTTGAGCGTGGAGC	AAGCAACACAGACTTCCCATCG
Alyli.0054s0075	AAGGTTAAAGACGCGGCAGA	TCTAGCATGACCTCCCGACA
Alyli.0282s0030	GGCTCCTGATGTTCCGAGAG	GGCCGAGCATCACTAAACCT
Alyli.0282s0030	CGCTGGATACAAGGACGGAA	CTTCACGCCAGTCTTCGTCT
AT2G25450	AAAAGTCCCGCGCATTTTCC	AAAAGTCCCGCGCATTTTCC
AT2G25450	CGACGGTGGTGATGATTCCA	CGCAACTACACTCTCTCGCA
AT2G30830	GATGTGGCCGTTACAAACCC	CCAATTCGCAGCTTGAGAGC
AT2G30840.1	GCTCTCCTTCAGCGAACTGG	AAACCAGGCTCAGGACAAGG
Bostr.26326s0118	ATGACGGGAGATTACGACCG	TCACCACTGTCGAGGAAAGC
Brara.D01848	CGAGTCCACGGTCACAAGAA	AGGTATCTCTCCAGCTCGCA
Brara.D01849	TTTGACCGTGCCGATGAAGT	AACACGCCACCTCCCATATC
Brara.K01198	ACTCCGGGTGCTCTTATCCT	GCGGTGGTGTCTCTGTACTT
Crahi.0578s0006.1	TTAACCACGGCTTCCCACTC	ACTCCAGCATGACTTCCCCA
Desop.0248s0927	CTGCTGCGAGTTGGAGAGAT	CAGTGGCCGAGCATCACTAA
Isati.0111s0004.1	GCACTTCGGTTGCGTCTTTC	GAGGCTTCCGCATTGGTTTC
Isati.0576s0034.1	CCGCGCATTTTCCATAACCC	TTAAGCTCCGCAACCACACT
Isati.0905s0036.1	TTTCAATGGTGGCGTGTTCG	CTGGCAAGTCCTCTGCCTTT
Mamar.0031s0026-	TCACGACCACATTGGAGGAC	CGCGGCTCTTCACCTGTATT
Myper.0004s0500	ATGAGGTTAGGCGTGAAGGG	ATCGAACACGCCACCATTGA
Myper.0004s0502.1	CCGTGCCGATGAGTTGAAAG	GCGTGATCGTGGAATCGAAC
Stapi.4447s0001.1	TTTATGACTCTCCGGCTGCG	CCGAGTGTTAGGTCTGGCTC
Stapi.7214s0002.1	ATGAAGATCGGCGTGAAGGG	CGCAGCCGGAGAGTCATAAA
Caamp.0105s0129	GGAACATTTGACCGTGCCTG	CATGCGCGGGATTTTTGTGA
Braju.03G405000	ACTCCGGGTGCTCTTATCCT	GCGGTGGTGTCTCTGTACTT
Braju.04G167000	CGAGTCCACGGTCACAAGAA	AGGTATCTCTCCAGCTCGCA
Braju.04G167100	GCGCACTTCGATTGCATCTT	GCGACATAGTGGTTGGAAGC
Braju.04G167200	ATATGGGAGGTGGCGTGTTC	CGAACTTCTCCATGGCGTCT
Dtenuisiliqua.00017018	GTCCAACAACGCTTGCACTT	GTCCAACAACGCTTGCACTT
Dtenuisiliqua.00032667	CTGAGCCAGACCGAACACTC	AACGCTTGCACTTGTTGGAG
Eusyr.0020s0284	GCCGGAATCACACAAATCCC	GACGGTGGATTCGAACATGC
Eusyr.0020s0287	GCCAGACCTAACACTAGGCG	AAAACCCTATGCTCCACGCT
Hincana00039109	TGGAACACCGAGTTTTGGCT	AACGCTTGCACTTGTTGGAG
Msinaica.00026727	GAGCCAGACCTAACACTCGG	GCACATAGTGGTTGGAGGCT
Mspinosa.00027863	CCCACCTTGTCCTGAACCTG	GCGGGTGGTTACTCCCATTT
Rsativus.108851740	CCACGCATTTTCCACAACCC	CGAATACGCCACCTCCGAAA
Sialb.1587s0010	CTGAGCCAGACCGAACACTT	CGTCCGGATTAACGGGAACA
